# A Conductive Dinuclear Cuprous Complex Mimicking the Active Edge Site of the Copper(100)/(111) Plane for Selective Electroreduction of CO_2_ to C_2_H_4_ at Industrial Current Density

**DOI:** 10.34133/research.0008

**Published:** 2022-12-21

**Authors:** Jin-Meng Heng, Hao-Lin Zhu, Zhen-Hua Zhao, Da-Shuai Huang, Jun-Yi Li, Pei-Qin Liao, Xiao-Ming Chen

**Affiliations:** MOE Key Laboratory of Bioinorganic and Synthetic Chemistry, School of Chemistry, Sun Yat-Sen University, Guangzhou 510275, China.

## Abstract

Inorganic solids are a kind of important catalysts, and their activities usually come from sparse active sites, which are structurally different from inactive bulk. Therefore, the rational optimization of activity depends on studying these active sites. Copper is a widely used catalyst and is expected to be a promising catalyst for the electroreduction of CO_2_ to C_2_H_4_. Here, we report a conductive dinuclear cuprous complex with a short Cu···Cu contact for the electroreduction of CO_2_ to C_2_H_4_. By using 1*H*-[1,10]phenanthrolin-2-one and Cu(I) ions, a dinuclear cuprous complex [Cu_2_(ophen)_2_] (Cuophen) with a remarkable conductivity (3.9 × 10^−4^ S m^−1^) and a short intramolecular Cu···Cu contact (2.62 Å) was obtained. Such a short Cu···Cu contact is close to the distance of 2.54 Å between 2 adjacent Cu atoms in the edge of the copper(100)/(111) plane. Detailed examination of Cuophen revealed a high activity for the electroreduction of CO_2_ to C_2_H_4_ with a Faradaic efficiency of 55(1)% and a current density of 580 mA cm^−2^, and no obvious degradation was observed over 50 h of continuous operation. Comparing the properties and mechanisms of Cuophen and 2 other copper complexes with different Cu···Cu distances, we found that the shorter Cu···Cu distance is conducive not only for a *CO species to bridge 2 copper ions into a more stable intermediate transition state but also for C–C coupling.

## Introduction

Copper is one of the most widely used catalysts in the industry today. Because copper has a higher CO binding energy, it contributes to the hydrogenation of CO and converts CO_2_ into industrial products, such as hydrocarbons and ethanol [1–6]. In its metallic nanoparticle form, copper has further demonstrated promise as a promising catalyst for the electrochemical CO_2_ reduction reaction (eCO_2_RR) into hydrocarbons such as CH_4_, C_2_H_4_, and CH_3_CH_2_OH [7–11]. Similar to many inorganic solids, the catalytic activity of metallic copper is localized to rare surface sites, whereas the bulk material is relatively inert. Previous studies on the catalytic properties of metallic copper show that the Cu(100) crystal plane and the Cu(100)/Cu(111) interface are favorable for ethylene production. Although the performance of the Cu(100)-rich film and the Cu(100)/Cu(111) interface is very good, the specific catalytic active center is still controversial [12–18]. Because of the anisotropy of the crystalline material, it is very difficult to fully realize the pure (100) or (111) plane of metallic copper, thus making it hard to extensively study the catalytic site.

Mimicking copper surface with dicopper active sites for studies of catalytic performances should be helpful to understand the mechanism of copper catalyzing conversion of CO_2_ to ethylene, especially the effect of the Cu···Cu distance on the selectivity of C_2_ products, and provide effective ideas for a more precise design of catalytic materials. At present, despite the fact that small molecular complexes and metal-organic frameworks with dicopper active sites mimicking the Cu(100)/(111) crystal plane have been reported for the electrocatalytic reduction of CO_2_ to ethylene [19,20], the effect of the Cu···Cu distance on the selectivity of C_2_ products is still unclear. The performance of these materials with dicopper active sites is lower than that of the Cu(100) crystal plane and the Cu(100)/Cu(111) interface [21–25]. The poor performance of these dicopper active sites might be ascribed to their Cu···Cu distances (ranging from 2.78 to 3.77 Å) that are significantly longer than those of adjacent Cu–Cu in metallic copper. In addition, the conductivities of the dicopper catalysts for the eCO_2_RR are usually low; thus, the current density is rather limited. Obviously, an electrocatalyst simultaneously having a good conductivity and a short Cu···Cu distance should be a suitable candidate for electrocatalytic reduction of CO_2_ to ethylene.

**Fig. 1.**
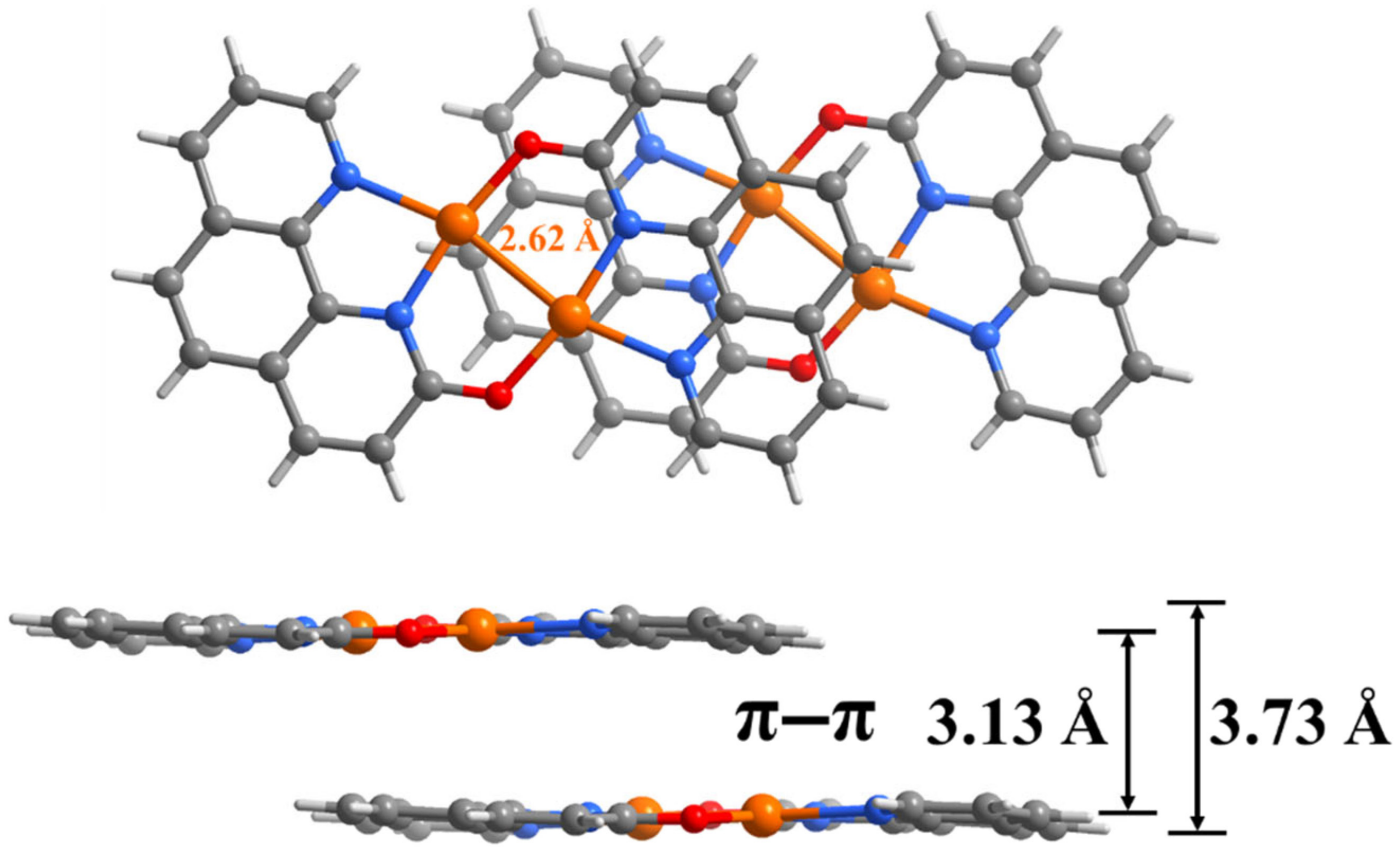
Top (top) and side (bottom) views of Cuophen.

[Cu_2_(ophen)_2_] (Cuophen, Hophen = 1*H*-[1,10]phenanthrolin-2-one) is a discrete cuprous complex, which stacks via strong face-to-face *π–π* stacking interaction to form a one-dimensional chain (Fig. 1 and Table S1) [26–29]. The largely conjugated and strong *π–π* stacking structure should be beneficial for the conductivity. In Cuophen, each Cu(I) ion adopts a trigonal geometry, being coordinated by 2 nitrogen atoms from one ophen^–^ ligand and one deprotonated hydroxyl oxygen from another ophen^–^ ligand. A pair of T-shaped coordinated Cu(I) ions share a short intramolecular metal–metal contact (2.62 Å) to accomplish square–planar coordination spheres, forming a dimeric structure. The Cu···Cu distance in Cuophen is very close to that (2.54 Å) of the Cu–Cu at the edge site of Cu(100) (Fig. S1), which is expected to be favorable for the electrochemical reduction of CO_2_ to C_2_H_4_. In addition, there is a strong face-to-face *π*–*π* stacking interaction between molecules, and the intermolecular Cu···Cu distance is 3.60 Å, which is similar to the interaction between surface copper atoms and sublayers of metallic copper. Therefore, Cuophen should be able to mimic Cu(100)/Cu(111) better than other small molecule complexes with metal-organic frameworks that have been reported. In this work, Cuophen was employed to investigate the performance and mechanism for selective conversion of CO_2_.

## Results and Discussion

The ligand Hophen was synthesized by a solvothermal method [30,31], verified by ^1^H nuclear magnetic resonance (^1^H NMR) spectroscopy (Fig. S2). Solvothermal reaction of CuCl and Hophen at 160 °C for 96 h gave black microcrystalline powder of Cuophen (Fig. S3). The thermogravimetric curve of Cuophen shows that the thermal decomposition temperature is 389 °C, indicating an excellent stability (Fig. S4). The purity of Cuophen was confirmed by powder x-ray diffraction pattern (Fig. S5), scanning electron microscopy images (Fig. S6), and transmission electron microscopy (TEM) images (Fig. S7). Scanning transmission electron microscopy (STEM)–x-ray dispersive analysis (Fig. S8) confirmed that the elements Cu, C, O, and N are uniformly distributed. Aberration-corrected high-angle annular dark field (HAADF)-STEM was performed on the direct observation of Cuophen. Interestingly, the dots of pairwise combinations can be seen (Fig. S9A). Since Cu atoms are much heavier in comparison to C, N, and O atoms, the bright dots should be Cu atoms. The distance between the dual dots was estimated to be 2.62 Å (Fig. S9B), which is the same as that obtained from the crystal structure. The x-ray photoelectron spectroscopy (XPS) profile of Cu 2p and Cu LMM Auger spectrum analysis (Fig. S10) indicated that the Cu ions in Cuophen are +1 valent. Electrochemical impedance spectroscopy measurement of this compound shows an excellent conductivity (3.9 × 10^−4^ S·m^−1^) [29] due to its conjugated structure and strong *π–π* stacking interaction, which is beneficial for productive electron transfer in eCO_2_RR.

**Fig. 2.**
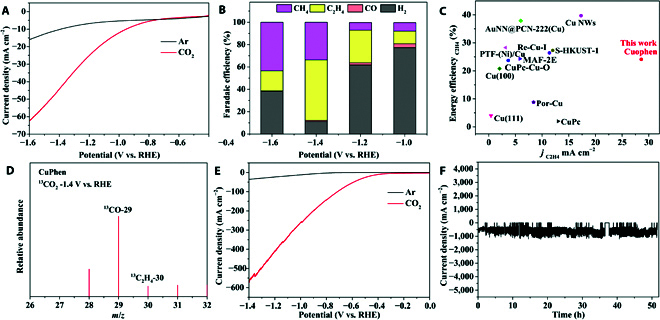
(A) Linear sweep voltammetry curves of Cuophen in a 0.1 M KHCO_3_ solution saturated with Ar and CO_2_, respectively. (B) FEs of different reduced products for Cuophen at the potentials of −1.0 to −1.6 V vs. RHE. (C) Energy efficiency as a function of partial current density on Cuophen in comparison with representative catalysts. (D) Mass spectrum of ^13^C_2_H_4_ recorded under ^13^CO_2_ atmosphere. (E) Linear sweep voltammetry curves of Cuophen in 1 M KOH solutions saturated with Ar and CO_2_, respectively. (F) Durability test of Cuophen at −1.4 V vs. RHE.

The eCO_2_RR performance of as-synthesized Cuophen was tested in a 3-electrode flow cell device with Pt foil as the counter electrode and Ag/AgCl electrode as the reference electrode. The working electrode was evenly coated with Cuophen catalyst on the gas diffusion layer modified carbon paper (Fig. S11). CO_2_ can easily pass through the carbon paper to contact with the electrolyte, but the electrolyte cannot escape through the carbon paper. The anion exchange membrane was used to separate the cathode and anode cell. Linear sweep voltammetry curves from −0.4 to −1.6 V versus reversible hydrogen electrode (RHE) were measured in a 0.1 M KHCO_3_ electrolyte saturated with CO_2_ and Ar, respectively (Fig. 2A). In the CO_2_ saturated solution, a more positive onset potential (−0.8 V vs. RHE) and a greater current density of 52 mA cm^−2^ at −1.4 V vs. RHE of Cuophen indicate the catalyst having favorable activity for eCO_2_RR (Fig. S12). Gas chromatography (Figs.S13 and S14) and ^1^H NMR spectroscopy were used to detect the gas and liquid products, respectively. As shown in Fig. S15, the gas-phase products of H_2_, CH_4_, C_2_H_4_, and CO were detected by gas chromatography. The test results show that the selectivity of products was greatly affected by the operation electrode potentials. At low potential, H_2_ was the most detected product and the maximum Faradaic efficiency (FE) was 77.3%. With the negative shift of electrode potential, the FE of H_2_ decreased gradually, while those of ethylene and methane increased gradually. At −1.4 V vs. RHE, the FE of C_2_H_4_ reached 55(1)% with a high current density of 52 mA cm^−2^ (Fig. 2B and Fig. S16). Such high current density may be ascribed to the excellent conductivity of Cuophen and the high activity of the dicopper sites. No liquid product was observed in the ^1^H NMR spectra (Fig. S17). Notably, as for the performance of eCO_2_RR towards C_2_H_4_ product in neutral electrolyte, Cuophen (energy efficiency [C_2_H_4_] of 25% and partial current density of 28.6 mA cm^−2^) is better than most of the Cu-based electrocatalysts such as Cu derivatives and Cu nanostructured materials (Fig. 2C and Tables S1 and S3). We also tested the performance of eCO_2_RR in a flow cell device with 1 M KOH as electrolyte. The FE of CH_4_ was 37% and that of C_2_H_4_ (55%) remained unchanged, while the current density was increased to 580 mA cm^−2^, being much higher than the commercially relevant current density of 300 mA cm^−2^ (Fig. 2E and Table S4). Furthermore, no obvious degradation was observed at 580 mA cm^−2^ and the FE of ethylene can still be maintained at 50% over 50 h of continuous operation, which further strengthens the practicability of Cuophen (Fig. 2F and Figs. S18 and S19). Notably, the partial current density of C_2_H_4_ reaches 319 mA cm^−2^, which is comparable to those of the 2 best materials—CuAl alloy [32] and CuPzH [33]—reported to date (Fig. S20). To verify the carbon origin of the product, ^13^CO_2_ isotopic investigation was further carried out. The peak of *m*/*z* = 30 assignable to ^13^C_2_H_4_ is clearly presented (Fig. 2D), confirming that the carbon atoms of C_2_H_4_ originate from the inlet CO_2_.

According to the powder x-ray diffraction patterns (Fig. S5), scanning electron microscopy and TEM images (Fig. S21), and XPS spectra (Fig. S10), no significant change of the structure was observed, and no Cu or Cu_2_O species were generated after electrocatalysis for 50 h, indicating the high stability of Cuophen, which is further demonstrated by the aberration-corrected HAADF-STEM (Fig. S22), the anodic stripping voltammograms (Fig. S23), and the time-dependent FEs of gaseous products (Fig. S24). To further confirm the structural stability of Cuophen, synchrotron-based x-ray absorption spectroscopy was also performed. The x-ray absorption near-edge structure spectra (Fig. S25) showed no significant changes in Cu *K*-edges before and after electrochemical treatment. The extended x-ray absorption fine structure spectra and fittings and wavelet transform spectra also remained unchanged before and after electrocatalysis, and there were no Cu clusters and Cu–Cu bond generated (Figs. S26 and S27), consistent with the XPS spectra and TEM image analysis. Through comprehensive consideration of the C_2_H_4_ efficiency, potential, current density, and stability, the performance of Cuophen is higher than most Cu-based catalysts (Fig. 2c, Fig. S20, and Tables S2 to S4), especially those with the polynuclear active sites (with the Cu···Cu distances ranging from 2.78 to 3.77 Å), highlighting the importance of the shorter Cu···Cu distance in Cuophen.

**Fig. 3.**
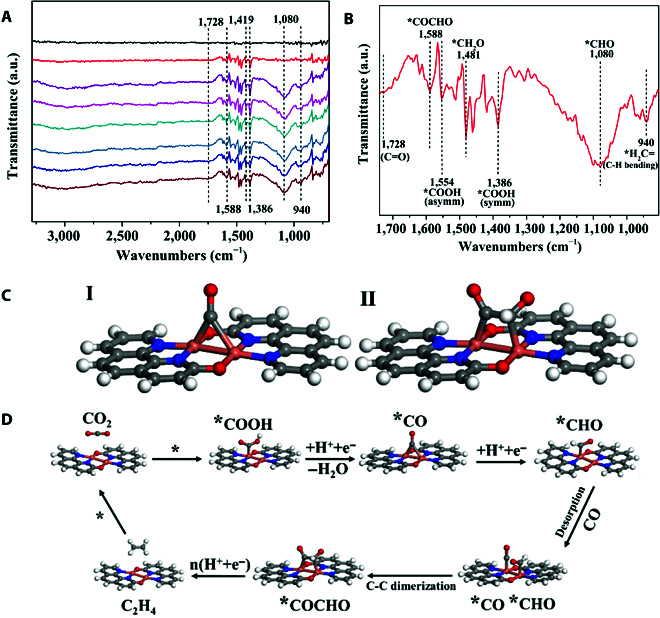
(A, B) Operando ATR-FTIR spectra of Cuophen during the eCO_2_RR. (C) Proposed eCO_2_RR intermediates of Cuophen: I, *CO; II, *COCHO. Color codes: carbon (gray), nitrogen (blue), oxygen (red), hydrogen (white), and copper (orange). (D) The possible reaction procedure of Cuophen catalyzing eCO_2_RR.

Operando attenuated total reflection infrared–Fourier transform infrared (ATR-FTIR) spectral measurements were conducted to study the mechanism of ethylene generation in the eCO_2_RR process. In Fig. 3A and B, the absorption peaks at 1,386 and 1,554 cm^−1^ can be attributed to symmetric and asymmetric stretches of the *COOH intermediates. Those at 1,588 and 1,728 cm^−1^ can be assigned to the asymmetric vibration of *COCHO and C=O stretching of carbonyl intermediates, respectively. In addition, the peaks at 1,080 and 1,481 cm^−1^ can be associated with the *CHO and *CH_2_O species, respectively. Most importantly, the peak that appeared at 940 cm^−1^ can be associated with the *CH_2_ intermediate. These are the key intermediates in the electroreduction of CO_2_ to C_2_H_4_ [34,35].

Based on ATR-FTIR spectroscopy, the catalytic mechanism of Cuophen in the eCO_2_RR process was simulated by the density functional theory calculations. It can be seen that the *CO intermediate (Fig. 3C), i.e., a CO bridging 2 Cu ions (adsorption enthalpy Δ*E* = −56 kJ mol^−1^), is more thermodynamically dominant than that of a CO coordinated with a single Cu ion (Δ*E* = −33 kJ mol^−1^). The bridging coordination mode of a *CO makes it easier to be activated and further reduced to form a *CHO with a lower Gibbs free energy barrier (Δ*G* = 0.45 eV) compared to that of the single coordination mode (Δ*G* = 0.98 eV). Then, the *CO and *CHO couple into a *COCHO species with a lower Gibbs free energy barrier (Δ*G* = −0.22 eV), which undergoes a multistep proton-coupled electron transfer process to produce C_2_H_4_. It can be seen that the shorter Cu···Cu distance is favorable not only for the formation of a more stable transition state of the bridging *CO intermediate but also for easier C–C coupling (Fig. 3D) [36].

**Fig. 4.**
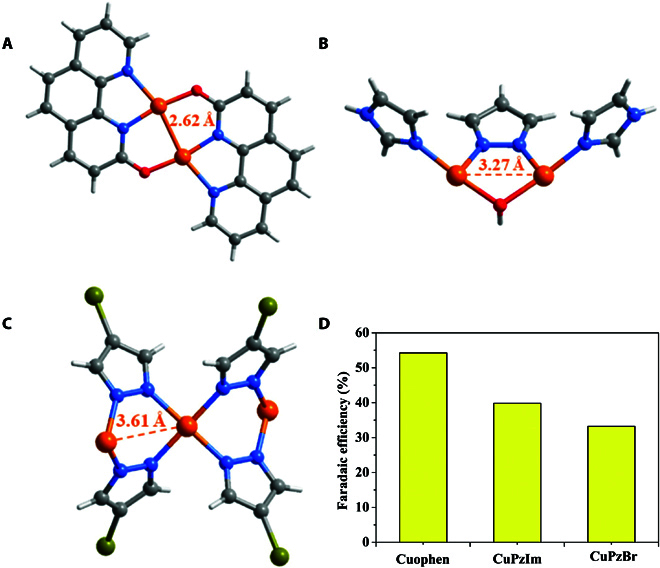
Comparison of the adjacent Cu···Cu distances of (A) Cuophen, (B) CuPzIm, and (c) CuPzBr, and (d) their Faradaic efficiencies for C_2_H_4_ in eCO_2_RR.

For further understanding the relationship between the Cu···Cu distance and selectivity of eCO_2_RR towards C_2_H_4_, 2 copper complexes, namely, trinuclear [Cu_3_(*μ*_3_-OH)(*μ*-pz)_3_(im)_3_]Br_2_ [37] (CuPzIm, Hpz = pyrazole, im = imidazole) and polymeric [Cu(4-BrPz)_2_] [33] (CuPzBr, 4-BrHPz = 4-bromopyrazole) with the adjacent Cu···Cu distances of 3.27 and 3.61 Å, respectively, were also synthesized for comparison (Fig. S28). Under the same conditions, the FEs of C_2_H_4_ are in the sequence of Cuophen (55%) > CuPzIm (40%) > CuPzBr (33%), and the Cu···Cu distances are in a reverse sequence of Cuophen (2.62 Å) < CuPzIm (3.27 Å) < CuPzBr (3.61 Å) (Fig. 4). It can be seen that Cuophen has a significantly higher FE(C_2_H_4_) than CuPzIm and CuPzBr. Density functional theory calculations show that the energy barriers for the coupling of *CO with *CHO to form *COCHO species by CuPzIm and CuPzBr are 0.03 and 0.16 eV, respectively, which are higher than that (−0.22 eV) by Cuophen, indicating that the shorter Cu···Cu distance is more favorable for the formation of the key intermediate *COCHO to produce C_2_H_4_ product.

## Conclusion

To conclude, a conductive dinuclear cuprous complex exhibits an impressive performance for the electroreduction of CO_2_ to C_2_H_4_ products. As revealed by the mechanism study, such high performance may be ascribed to its remarkable conductivity and the intramolecular Cu···Cu distance in this complex being very close to that of the copper(100)/(111) crystal plane. This work, for the first time, demonstrates concrete evidence for the important effect of the Cu···Cu distance on the selectivity of C_2_ products and may provide a new clue to understand and design electrocatalysts for conversion of CO_2_ into high-valued C_2+_ hydrocarbons.

## Materials and Methods

Cuprous chloride (CuCl, 97%, Aladdin), 1*H*-1,10-phenanthrolin-2-one (Hophen, 95%, HWRK), *N*,*N*-dimethylacetamide (DMA, 98.5%, GHTECH), pyrazole (C_3_H_4_N_2_, 98%, D&B Biological), imidazole (C_3_H_4_N_2_, 99%, D&B Biological), cupric bromide (CuBr_2_, 98.5%, Alfa Aesar), cupric nitrate (Cu(NO_3_)_2_·3H_2_O, 99%, Adamas), 4-bromopyrazole (C_3_H_3_BrN_2_, 98%, Acmec Biochemical), acetone (C_3_H_6_O, 99.5%, Guangzhou), potassium bicarbonate (KHCO_3_, 99.9%, Macklin), isopropyl alcohol (C_3_H_8_O, 99.5%, Aladdin), and potassium hydroxide (KOH, 85.0%, Guangzhou) were used in the study.

### Synthesis of Cuophen

A mixture of CuCl (0.099 g, 1.0 mmol), Hophen (0.196 g, 1.0 mmol), DMA (10.4 ml), and acetone (1.3 ml) was put in a 15-ml stainless steel vessel and heated at 160 °C for 96 h and then cooled to room temperature. The mixture was washed several times with DMA and dried at 50 °C. Large black crystals were obtained (yield ca. 45%). Elemental analysis calcd (%) for C_24_H_14_Cu_2_N_4_O_2_: C 55.70, H 2.73, and N 10.83; found: C 55.30, H 2.95, and N 10.73.

### Synthesis of CuPzIm

A: Pyrazole (0.091 g, 1.3 mmol) and imidazole (0.082 g, 1.2 mmol) were dissolved in 20 ml of deionized water; B: CuBr_2_ (1.2 mmol, 0.268 g) was dissolved in 20 ml of deionized water. After pouring B into A and mixing well, the mixture was transferred to a 150-ml Pyrex vial and stirred at 30°C for 3 h. After filtration and washing with deionized water and drying, purple powder was obtained (yield ca. 60%).

### Synthesis of CuPzBr

Cu(NO_3_)_2_·3H_2_O (0.024 g, 0.1 mmol) and 4-bromopyrazole (0.044 g, 0.3 mmol) were dissolved in DMA:H_2_O = 10:5 (DMA = 2 ml, H_2_O = 1 ml) mixed solution by ultrasonication. The solution was transferred to a 20-ml Pyrex vial, sealed at 100 °C for 72 h, and then cooled at a rate of 15 °C/h. After filtration and washing with deionized water and drying, purplish brown powder was obtained (yield ca. 30%).

## Data Availability

The data used to support the findings of this study are included within the article.

## Supplementary Materials

Supplementary Materials Supplementary Information Text Fig. S1. Structures of (A) Cuophen and (B) metallic copper. Fig. S2. ^1^H-NMR spectrum of Hophen (300 MHz, DMSO). Fig. S3. Photographs of bulk black crystals (A) and a single crystal (B) of Cuophen. Fig. S4. Thermogravimetric curve of Cuophen. Fig. S5. PXRD patterns of Cuophen before and after electrocatalysis. Fig. S6. SEM image of Cuophen. Fig. S7. TEM image of Cuophen. Fig. S8. (A) TEM image of Cuophen catalyst. Fig. S9. (A) Aberration-corrected HAADF-STEM image of Cuophen. Fig. S10. (A) XPS spectra of Cuophen before and after the electrocatalysis. Fig. S11. Schematic illustration of a flow cell device. Fig. S12. *i*–*t* curves of Cuophen for electrocatalytic CO2 reduction at the potentials of −1.0 to −1.6 V vs. RHE in 0.1 M KHCO3. Fig. S13. GC profiles of the standard gases with different concentrations: (A) 10 ppm, (B) 50 ppm, (C) 100 ppm, and (D) 500 ppm. Fig. S14. The standard curves on GC. (A) C_2_H_4_, (B) CH_4_, (C) H_2_, and (D) CO. Fig. S15. GC profiles of Cuophen catalyzing eCO2RR with different potentials: (A) −1.0 vs. RHE, (B) −1.2 vs. RHE, (C) −1.4 vs. RHE, and (D) −1.6 vs. RHE. Fig. S16. FEs of different reduced products for Cuophen after the electrocatalysis at the potentials of −1.0 V to −1.6 V vs. RHE. Fig. S17. ^1^H NMR spectra of the liquid phase before (A) and after (B) electrocatalysis of Cuophen. Fig. S18. GC profiles of Cuophen after the electrocatalysis at −1.4 V vs. RHE for 50 h with different potentials: (A) −1.0 vs. RHE, (B) −1.2 vs. RHE, (C) −1.4 vs. RHE, and (D) −1.6 vs. RHE. Fig. S19. (A) FEs of CH_4_, C_2_H_4_, CO, and H_2_ for Cuophen after the electrocatalysis. Fig. S20. Energy efficiency as a function of partial current density on Cuophen with 1 M KOH in comparison with representative catalysts. Fig. S21. (A) SEM and (B) TEM images of Cuophen after the electrocatalysis. Fig. S22. (A) Aberration-corrected HAADF-STEM image of Cuophen after the electrocatalysis. Fig. S23. Anodic stripping voltammograms obtained from Cuophen-modified glassy carbon electrode when the potential was held at −1.4 V vs. RHE in a CO_2_-saturated 0.1 M KHCO_3_ aqueous solution (scan rate: 50 mV s^−1^). Fig. S24. Faradaic efficiencies of gaseous products in the first 90 min. Fig. S25. X-ray absorption spectroscopy characterization of catalyst and references. Fig. S26. (A) K’ space EXAFS spectrum and fits, (B) R’ space EXAFS spectrum and fits, and (C) Wavelet transform (WT) contour plots of EXAFS for the Cuophen before eCO_2_RR. Fig. S27. (A) K’ space EXAFS spectrum and fits, (B) R’ space EXAFS spectrum and fits, and (C) Wavelet transform (WT) contour plots of EXAFS for the Cuophen after eCO_2_RR. Fig. S28. PXRD patterns of (A) CuPzIm and (B) CuPzBr. Table S1. Crystal data and structure refinement for Cuophen. Table S2. Comparison of the electrocatalytic performances of representative Cu-based catalysts. Table S3. Performance comparison of various catalysts for CO_2_ electroreduction to ethylene in 0.1 M KHCO_3_. Table S4. Performance comparison of various catalysts for CO_2_ electroreduction to ethylene in 1 M KOH. Table S5. EXAFS fitting parameters of the Cuophen and Cuophen-after sample measured under operando conditions. SI References
