# Supplementary material for: A Conductive Dinuclear Cuprous Complex Mimicking the Active Edge Site of the Copper(100)/(111) Plane for Selective Electroreduction of CO2 to C2H4 at Industrial Current Density
Source: Research (Wash D C). 2022 Dec 21;2022:0008. doi: 10.34133/research.0008 (PMC11407521; doi:10.34133/research.0008)
Supplement: Supplementary Materials — Supplementary Information Text Fig. S1. Structures of (A) Cuophen and (B) metallic copper. Fig. S2. 1H-NMR spectrum of Hophen (300 MHz, DMSO). Fig. S3. Photographs of bulk black crystals (A) and a single crystal (B) of Cuophen. Fig. S4. Thermogravimetric curve of Cuophen. Fig. S5. PXRD patterns of Cuophen before and after electrocatalysis. Fig. S6. SEM image of Cuophen. Fig. S7. TEM image of Cuophen. Fig. S8. (A) TEM image of Cuophen catalyst. Fig. S9. (A) Aberration-corrected HAADF-STEM image of Cuophen. Fig. S10. (A) XPS spectra of Cuophen before and after the electrocatalysis. Fig. S11. Schematic illustration of a flow cell device. Fig. S12. i–t curves of Cuophen for electrocatalytic CO2 reduction at the potentials of −1.0 to −1.6 V vs. RHE in 0.1 M KHCO3. Fig. S13. GC profiles of the standard gases with different concentrations: (A) 10 ppm, (B) 50 ppm, (C) 100 ppm, and (D) 500 ppm. Fig. S14. The standard curves on GC. (A) C2H4, (B) CH4, (C) H2, and (D) CO. Fig. S15. GC profiles of Cuophen catalyzing eCO2RR with different potentials: (A) −1.0 vs. RHE, (B) −1.2 vs. RHE, (C) −1.4 vs. RHE, and (D) −1.6 vs. RHE. Fig. S16. FEs of different reduced products for Cuophen after the electrocatalysis at the potentials of −1.0 V to −1.6 V vs. RHE. Fig. S17. 1H NMR spectra of the liquid phase before (A) and after (B) electrocatalysis of Cuophen. Fig. S18. GC profiles of Cuophen after the electrocatalysis at −1.4 V vs. RHE for 50 h with different potentials: (A) −1.0 vs. RHE, (B) −1.2 vs. RHE, (C) −1.4 vs. RHE, and (D) −1.6 vs. RHE. Fig. S19. (A) FEs of CH4, C2H4, CO, and H2 for Cuophen after the electrocatalysis. Fig. S20. Energy efficiency as a function of partial current density on Cuophen with 1 M KOH in comparison with representative catalysts. Fig. S21. (A) SEM and (B) TEM images of Cuophen after the electrocatalysis. Fig. S22. (A) Aberration-corrected HAADF-STEM image of Cuophen after the electrocatalysis. Fig. S23. Anodic stripping voltammograms obtained from Cuophen-m [file research.0008.f1.pdf]

Supporting Information

A Conductive Dinuclear Cuprous Complex Mimicking the Active Edge Site of Copper(100)/(111) Plane for Selective Electroreduction of CO_2 to C_2H_4 at Industrial Current Density

Jin-Meng Heng, Hao-Lin Zhu, Zhen-Hua Zhao, Da-Shuai Huang, Jun-Yi Li, Pei-Qin Liao,* Xiao-Ming Chen

MOE Key Laboratory of Bioinorganic and Synthetic Chemistry, School of Chemistry, Sun Yat-Sen University, Guangzhou 510275, China.

Correspondence should be addressed to Pei-Qin Liao; liaopq3@mail.sysu.edu.cn,

This PDF file includes:

Supplementary text
Figures S1 to S28
Tables S1 to S5
SI References

Supplementary Information Text

Characterizations:

Powder X-ray diffraction (PXRD) patterns were carried out on a Bruker D8 Advance diffractometer (Cu K*a*). Scanning electron microscopy (SEM) images were conducted on a SU8010 system. Transmission electron microscope (TEM) images were recorded by a FEI Tecnai G2 F30 working at 300 kV. X-ray photoelectron spectroscopy (XPS) measurements were carried out on an ESCALAB 250 spectrometer. Elemental Analysis (EA) was carried out on an Elementar Vario EL cube. ^{1}H Nuclear magnetic resonance (^{1}H NMR) measurement was performed on a Bruker AVANCE-400 MHz spectrometer using DMSO as a standard. Attenuated total reflection Fourier transform infrared spectroscopy (ATR-FTIR) spectra were recorded on a Nicolet 6700 spectrometer. Thermogravimetry (TG) analysis were recorded by a NETZSCH STA 2500 Regulus. X-ray absorption spectroscopy (XAS) measurements were colleted at the Singapore Synchrotron Light Source (SSLS) center, where a pair of chanel-cut Si (111) crystals was used in the monochromator.

Electrochemical measurements:

Working electrode preparation. The catalyst ink was prepared as follows: **Cuophen** (5 mg) and isopropyl alcohol (950 μL) were completely mixed. Ultrasound was performed within 10 degrees for 1 hour, and then Nafion solution (5 wt%, 50 μL) was added for another 30 minutes. Then 100 μL catalyst ink was dropped on the commercial gas diffusion layer modified carbon paper with 0.2 cm long and 1 cm wide (S = 0.2 cm^2), and dried completely before test.

Electrochemical measurements. Electrochemical tests were performed on a CHI660E electrochemical workstation. In this study, all the electrochemical experiments were carried out in a three-electrode flow cell setup using 0.1 M $KHCO_3$ aqueous electrolyte with Pt plate as the counter electrode and Ag/AgCl electrode (PK-1038) as the reference electrode. And the working electrode was coated with evenly dispersed **Cuophen** catalyst on the GDL hydrophobic carbon paper. The anion exchange membrane was used to separate the cathode and anode cell. All the electrode potentials were measured against the Ag/AgCl electrode and converted to reversible hydrogen electrode (RHE) based on the following equation:

$$E\ (vs\ \text{RHE}) = E\ (vs\ \text{Ag/AgCl}) + 0.197\ \text{V} + 0.059 \times \text{pH}$$

During the electrochemical test, the first step was to ensure that the observable gas diffusion electrolytic cell chambers were well sealed. Then, a peristaltic pump drove the electrolyte to clean all the pipes three times. The flow of CO_2 gas was controlled by the flow meter (Alicat Mc-100sscm-d) at a rate of 20 sccm through the cavity on the back of the carbon paper, and the peristaltic pump (LongerPump, BT100-2J) flowed through the cathode and anode cavities at a constant rate of 30 rpm. Anion exchange membrane

separated the two cavities to form a cathode chamber and an anode chamber. The Faraday efficiency of a certain gas product was calculated by the formula as follows:

$$\mathrm{FE}\ \% = \frac{nFxV}{j_{Tot}} \times 100$$

Where n = number of electrons transferred

F = Faraday's constant

x = mole fraction of product

V = total molar flow rate of gas

j_{Tot} = total current density

Products analysis:

The gas phase collection is also collected during the electrocatalytic reduction of carbon dioxide with three electrodes. During the test, carbon dioxide flows continuously through the cathode chamber at a constant flow rate of 20 SCCM, and the outlet is connected directly to the GC system (Agilent Technologies 7890B) to collect the gas products. The products were obtained at different constant voltages and analyzed by gas chromatography equipped with two flame ionization detectors (FID) and thermal conductivity detector (TCD). The contents of gas phase products were determined by the standard curves (Figure S13). The liquid products were configured with deuterium oxide (D_2O, 100 μl, 99.9%), dimethyl sulphoxide (DMSO, 100 μl), and electrolyte (500 μl). The liquid products were detected by ^{1}H NMR.

Computational methods:

Density functional theory (DFT) calculations were performed by the Materials Studio 5.5 package.

The structures of all intermediates in electrocatalysis were firstly optimized by Dmol^3 module. The generalized gradient approximation (GGA) with the Perdew-Burke-Ernzerhof (PBE) function and TS for DFT-D correction were employed to the calculation. The convergence tolerance of energy, force and displacement convergence were set as 1×10^{-5} Ha, 2×10^{-3} Ha and 5×10^{-3} Å, respectively. The core was treated using the effective core potential (ECP), and the electrons were treated by double numerical plus d-functions (DNP) basis set.

***In situ* attenuated total reflection infrared (ATR-FTIR) measurements:**

The experimental conditions of ATR-FTIR: Firstly, the catalyst ink was prepared according to the method in the **Working electrode preparation**. Then, 30 uL of catalyst ink was coated on GCE and baked dry with UV lamp. The working electrode was pressed tightly on the germanium crystal for infrared signal capture, and 6.5 mL of 0.1 M $KHCO_3$ solution was injected into the reaction cell. After purging with high-purity carbon dioxide

gas for 30 min, the data were collected at -1.4 V *vs.* RHE with the Ag/AgCl reference electrode and platinum wire counter electrode, and FTIR spectra were recorded manually at different times from 0 to 1500 s.

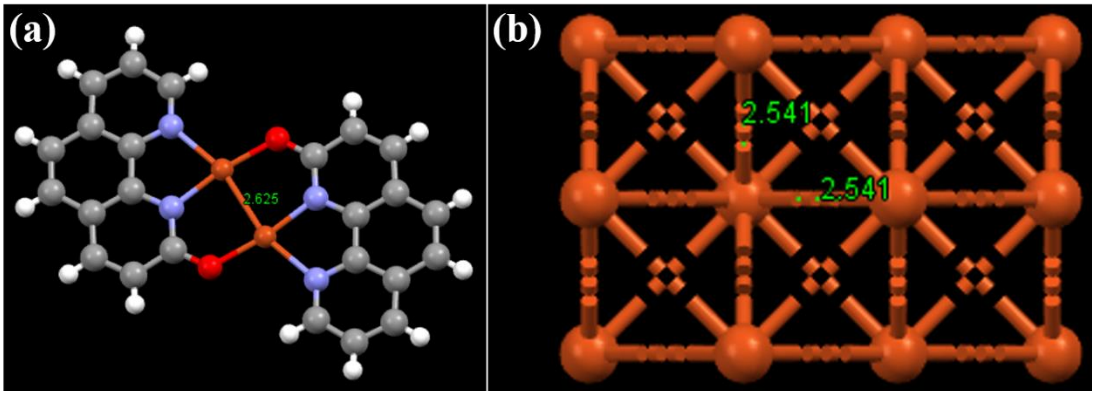

Fig. S1. Structures of (a) **Cuophen** and (b) metallic copper.

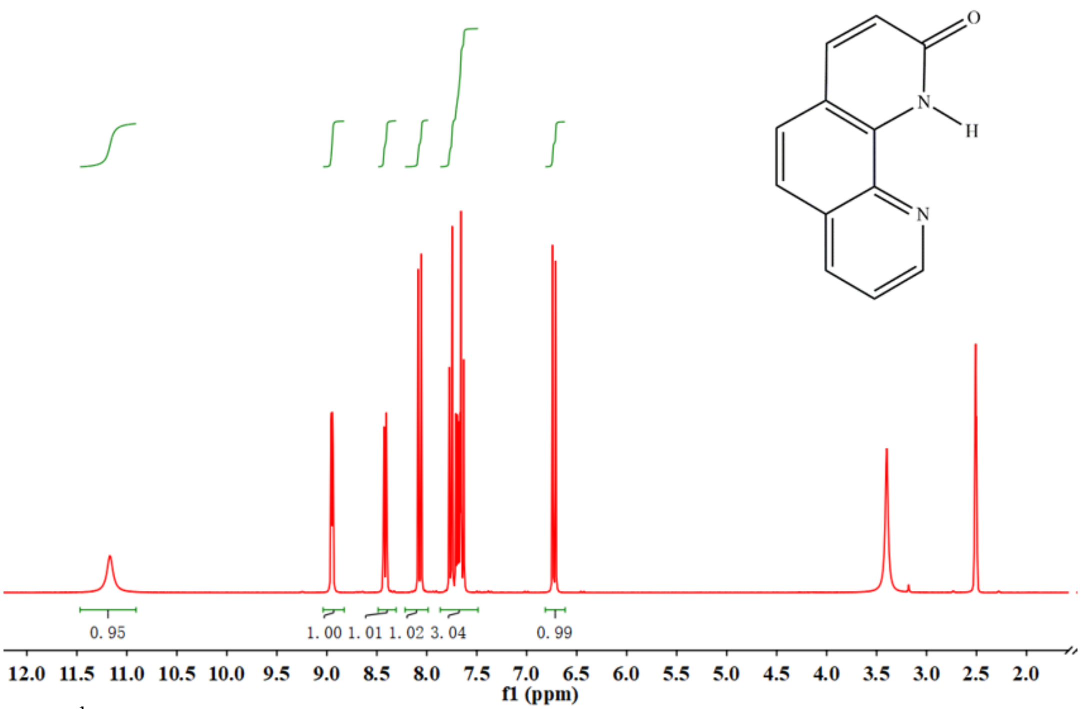

Fig. S2. ^{1}H-NMR spectrum of Hophen (300 MHz, DMSO). δ (ppm): 6.86(dd, 1 H), 7.53-7.64 (m, 3 H), 7.89 (d, 1 H), 8.21 (dd, 1 H), 8.92 (dd, 1 H) and 10.69 (broad s, 1 H).

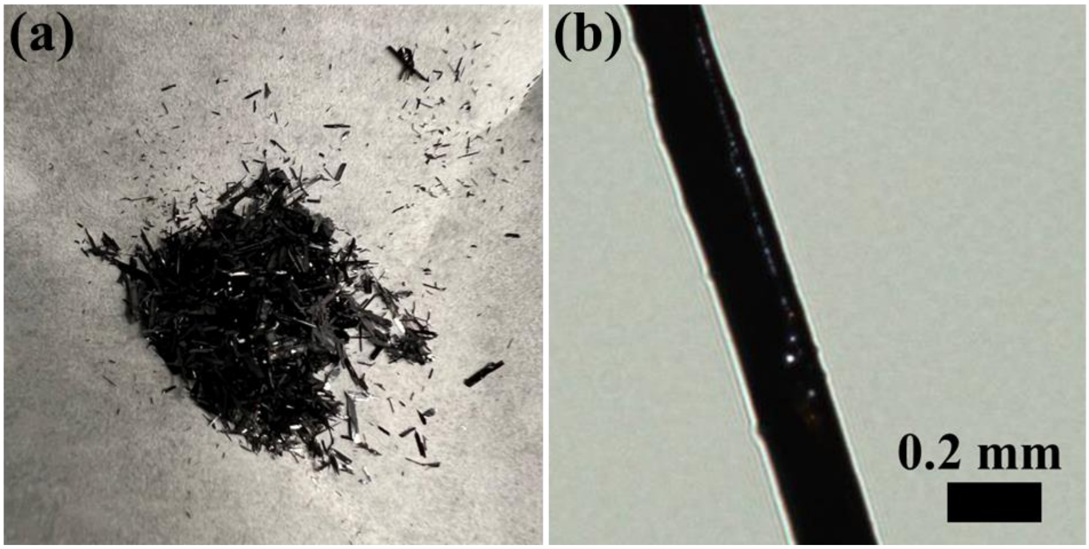

Fig. S3. Photographs of bulk black crystals (a) and a single crystal (b) of **Cuophen**.

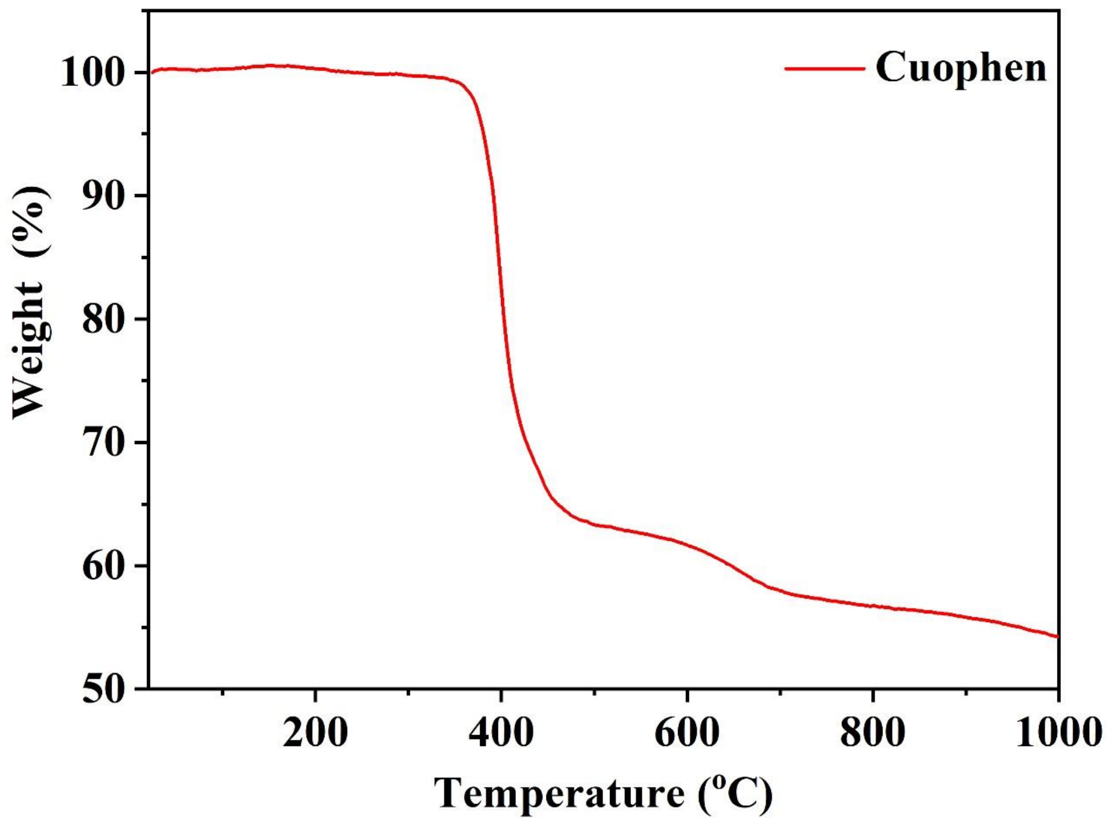

Fig. S4. Thermogravimetric (TG) curve of **Cuophen**.

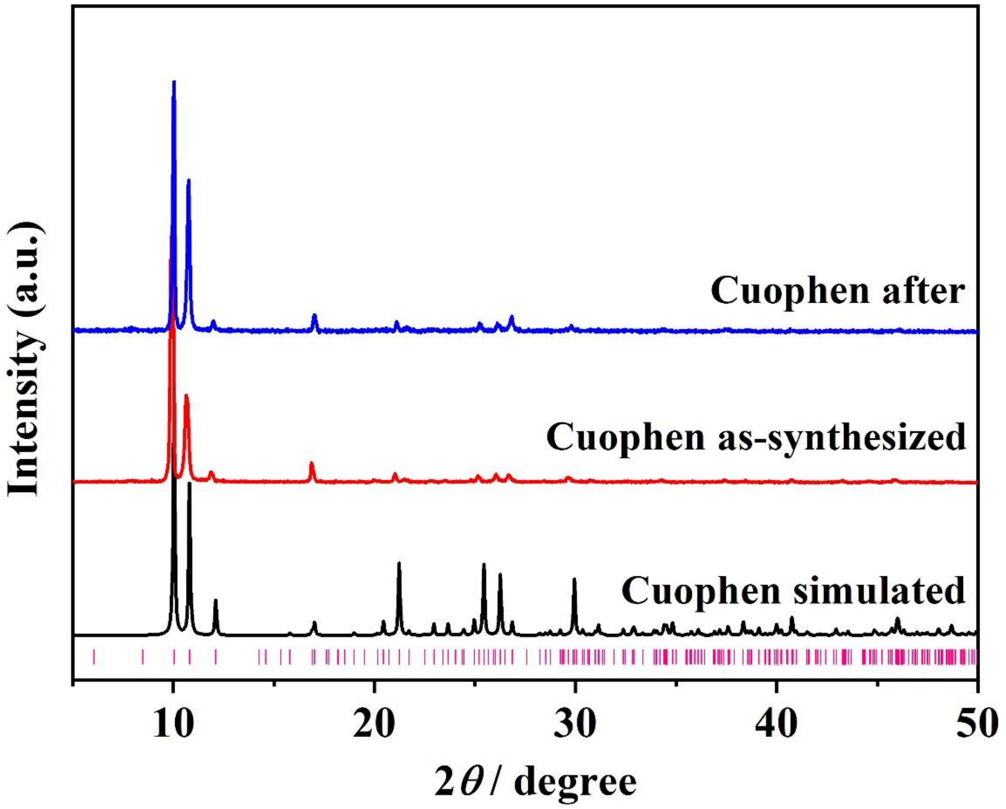

Fig. S5. PXRD patterns of **Cuophen** before and after electrocatalysis.

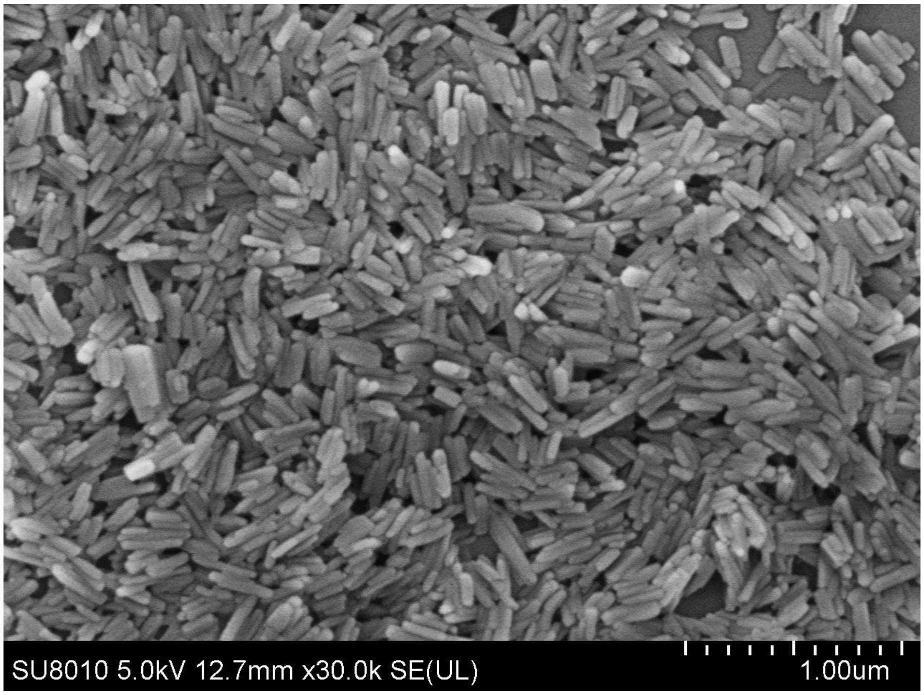

Fig. S6. SEM image of **Cuophen**.

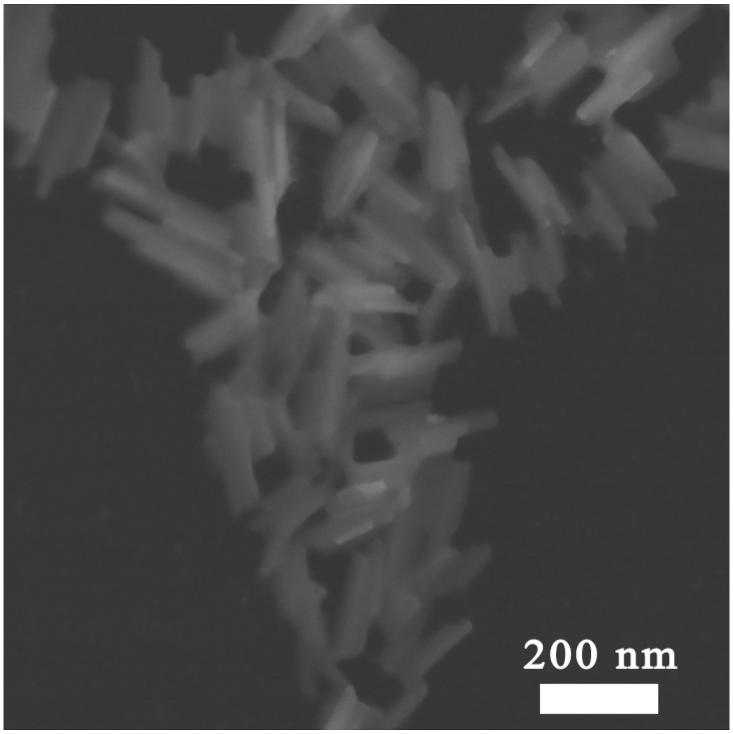

Fig. S7. TEM image of **Cuophen**.

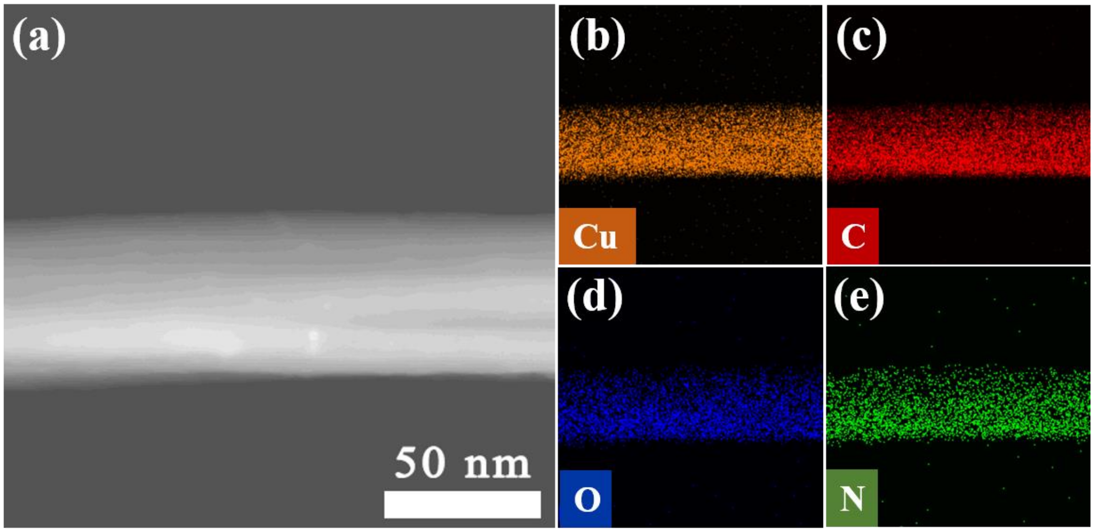

Fig. S8. (a) TEM image of **Cuophen** catalyst. (b, c, d, e) EDS elemental mapping images showing the homogenous distribution of all four elements of Cu, C, O, and N in **Cuophen**.

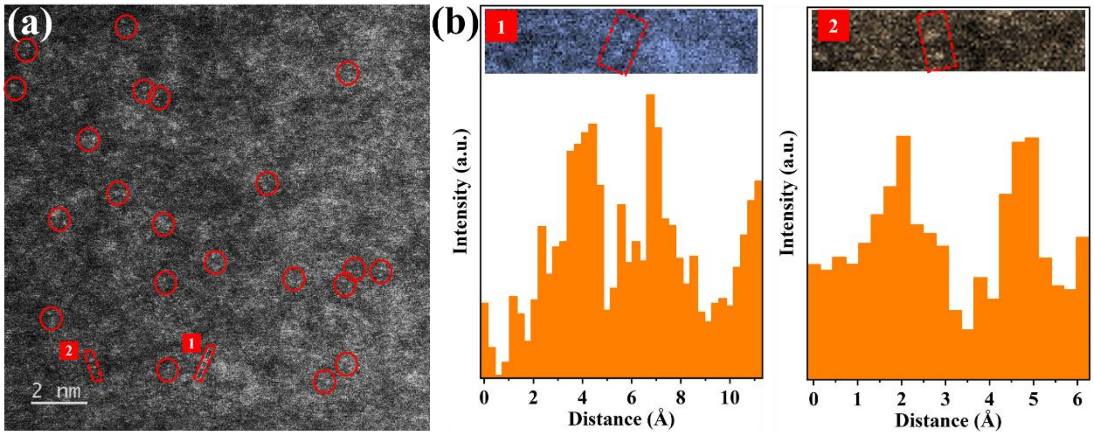

Fig. S9. (a) Aberration-corrected HAADF-STEM image of **Cuophen.** (b) Intensity profiles from the atomic sites 1 and 2 in (a).

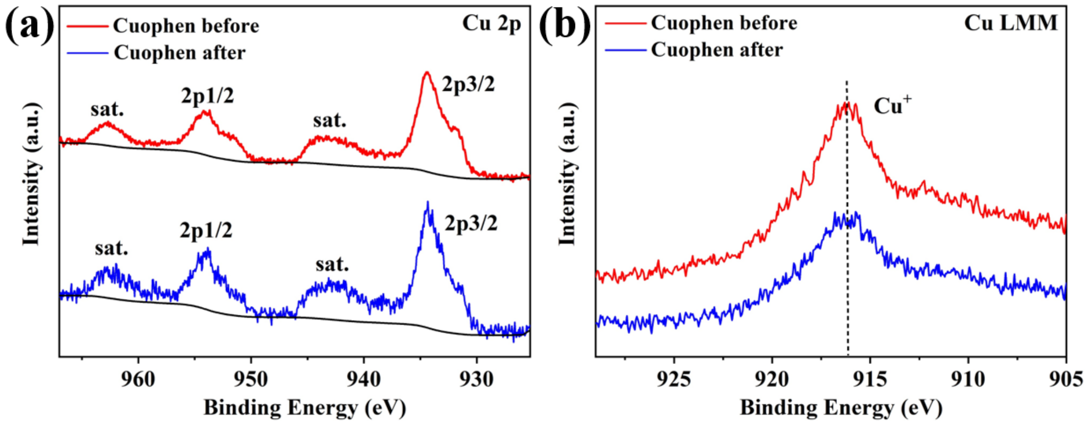

Fig. S10. (a) XPS spectra of **Cuophen** before and after the electrocatalysis. (b) Cu LMM spectra of **Cuophen** before and after the electrocatalysis.

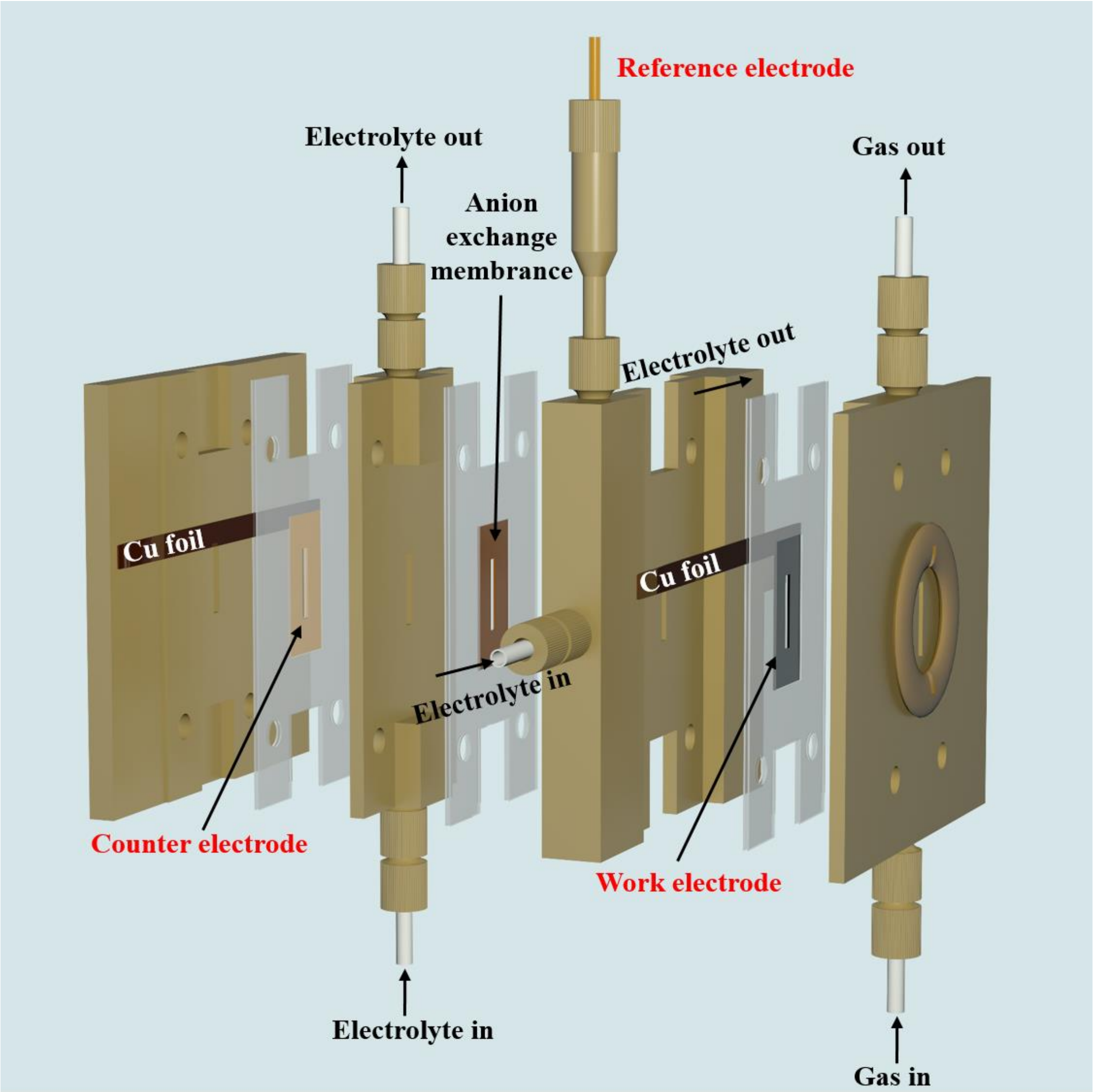

Fig. S11. Schematic illustration of a flow cell device.

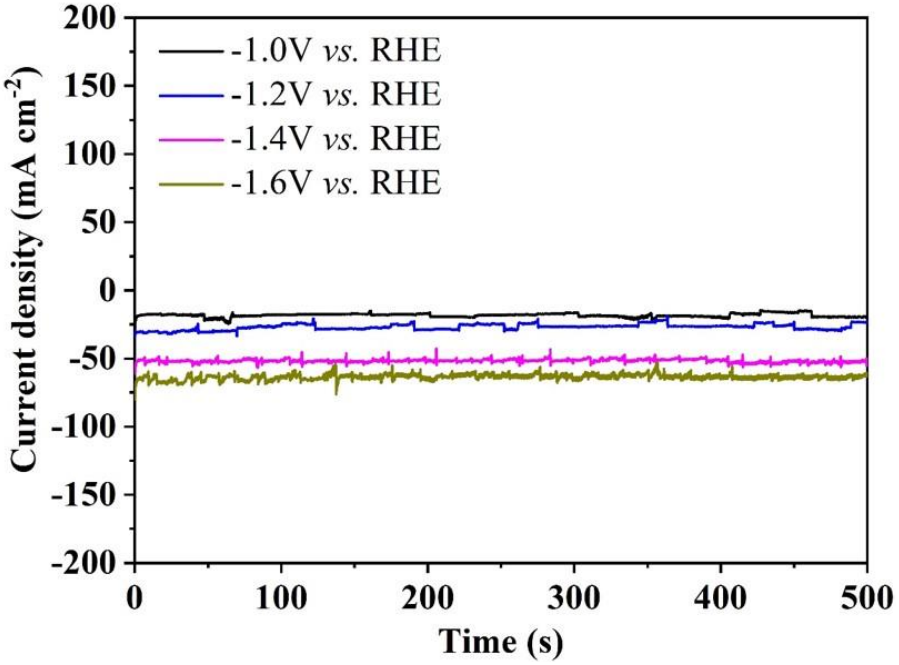

Fig. S12. *i-t* curves of **Cuophen** for electrocatalytic CO_2 reduction at the potentials of -1.0 to -1.6 V *vs*. RHE in 0.1 M $KHCO_3$.

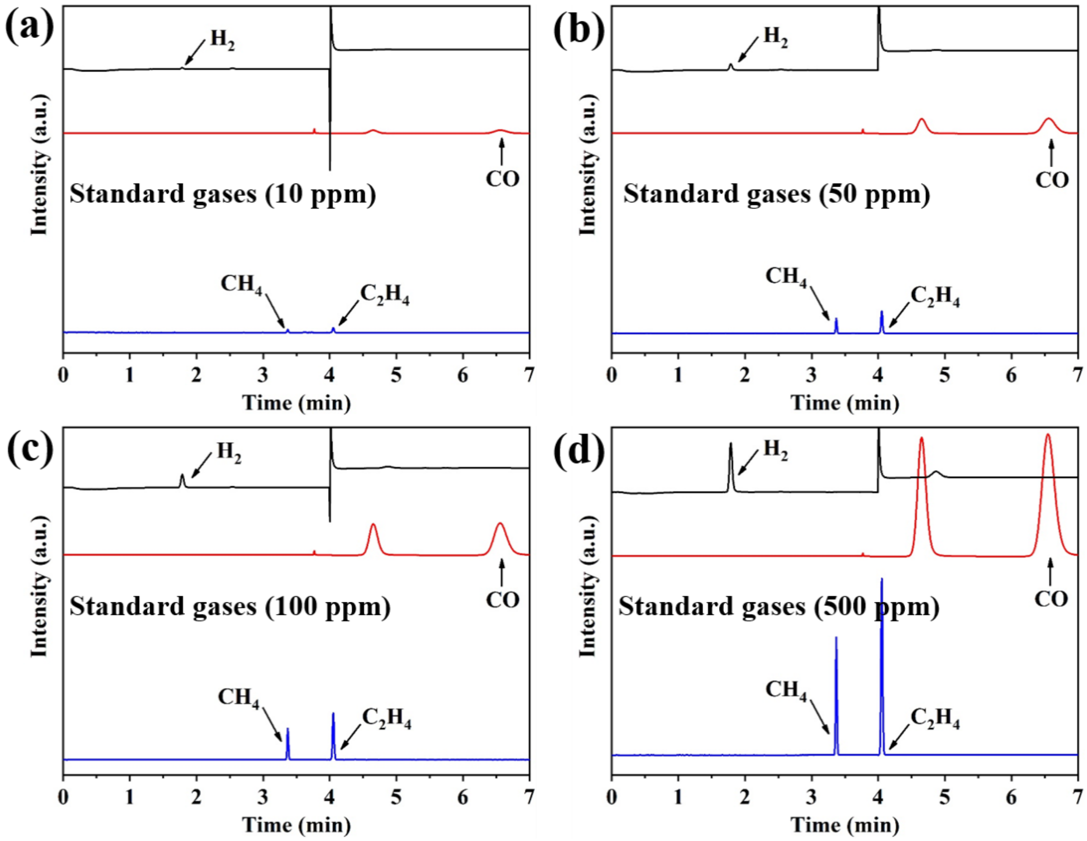

Fig. S13. GC profiles of the standard gases with different concentrations: (a) 10 ppm, (b) 50 ppm, 100 ppm and 500 ppm.

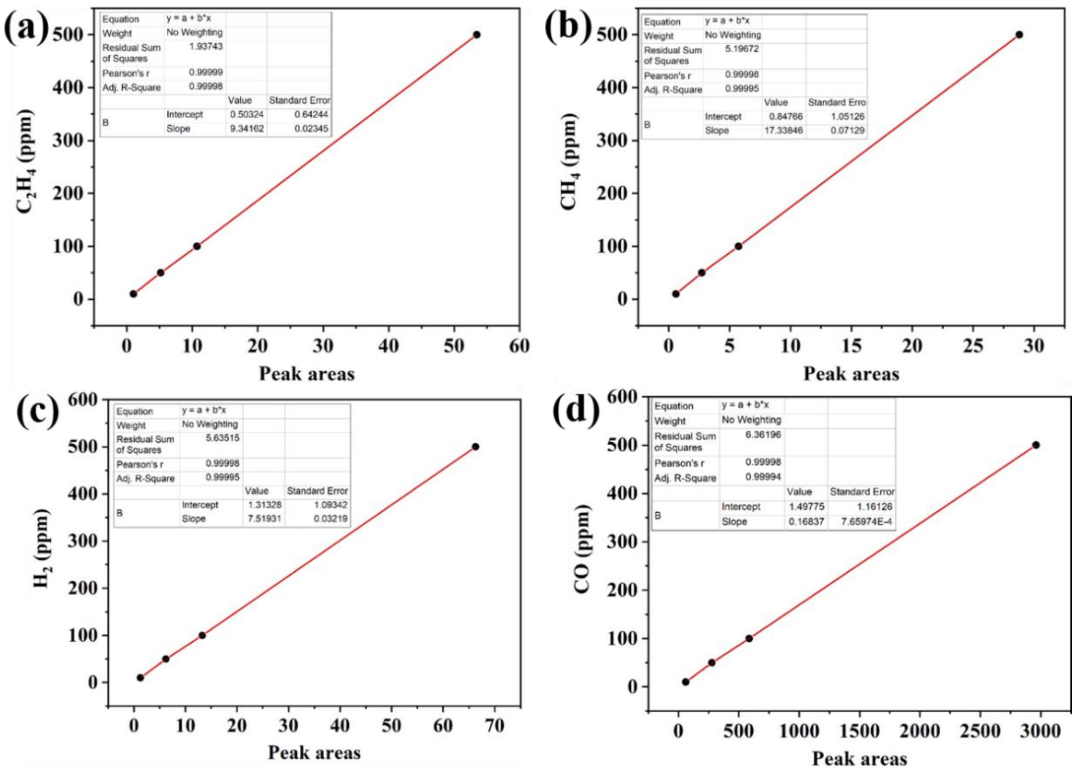

Fig. S14. The standard curves on GC. (a) C_2H_4, (b) CH_4, (c) H_2 and (d) CO.

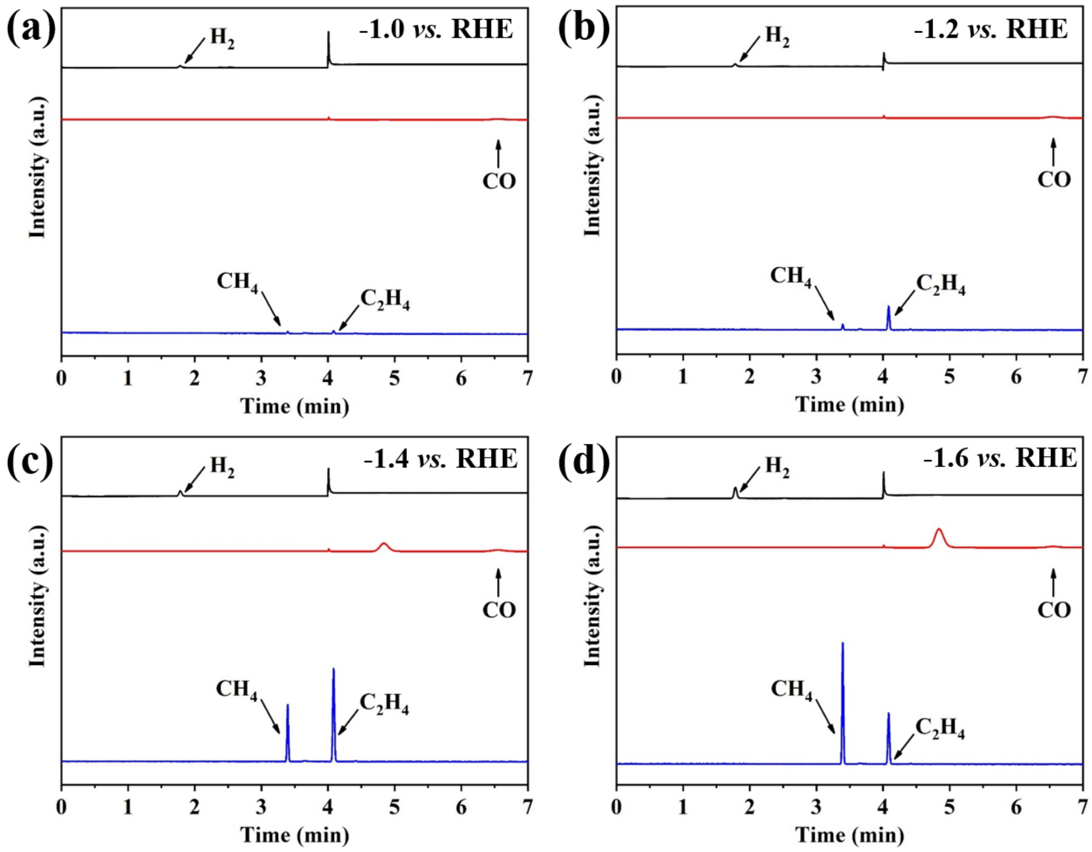

Fig. S15. GC profiles of **Cuophen** catalyzing eCO_2RR with different potentials: (a) -1.0 *vs.* RHE, (b) -1.2 *vs.* RHE, (c) -1.4 *vs.* RHE and (d) -1.6 *vs.* RHE.

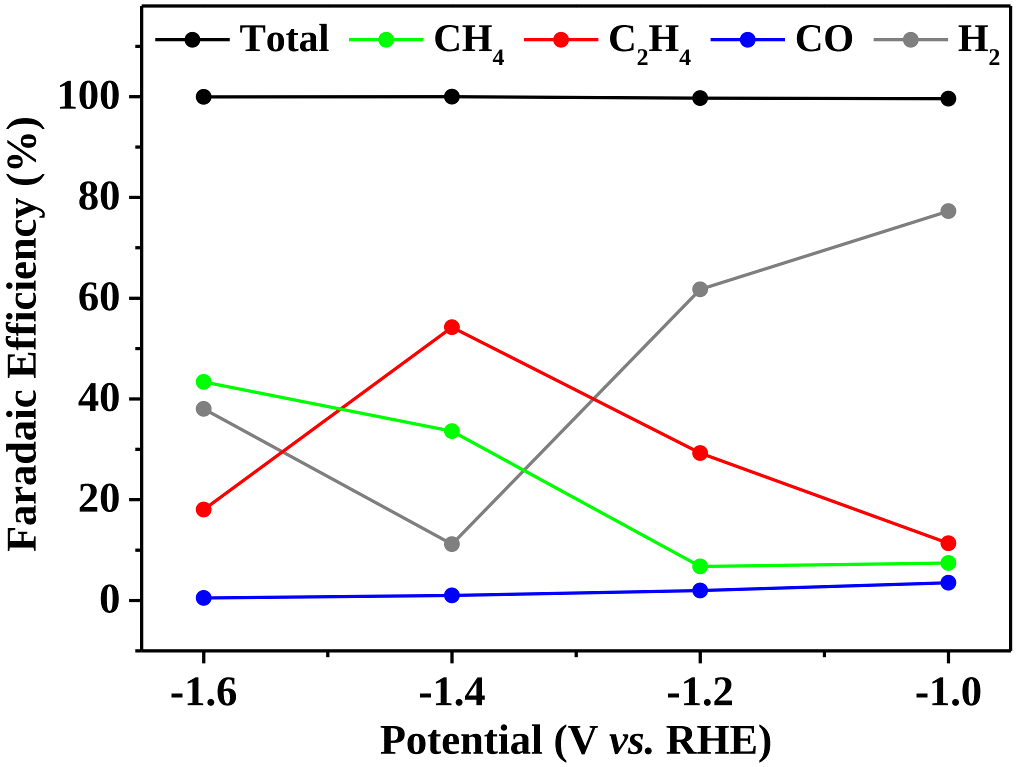

Fig. S16. FEs of different reduced products for **Cuophen** after the electrocatalysis at the potentials of -1.0 V to -1.6 V *vs.* RHE.

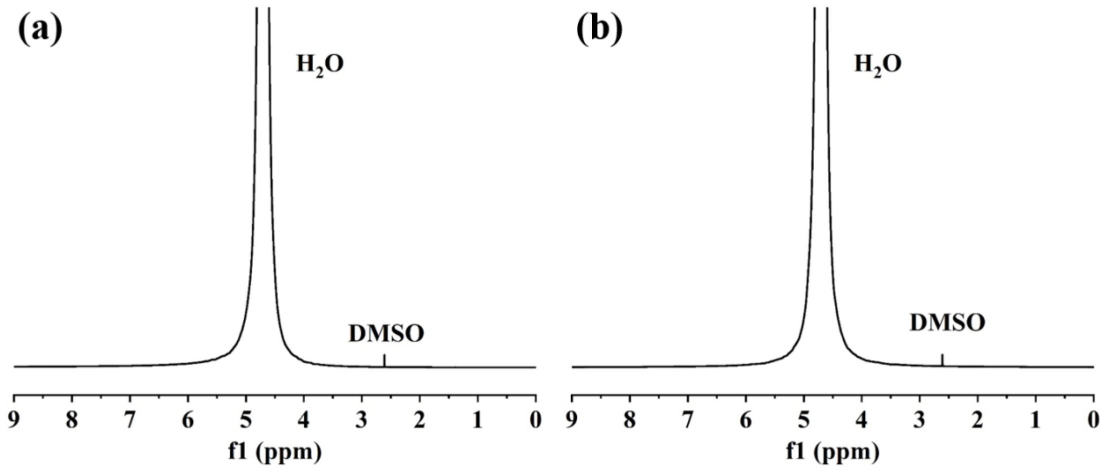

Fig. S17. ^{1}H NMR spectra of the liquid phase before (a) and after (b) electrocatalysis of **Cuophen**.

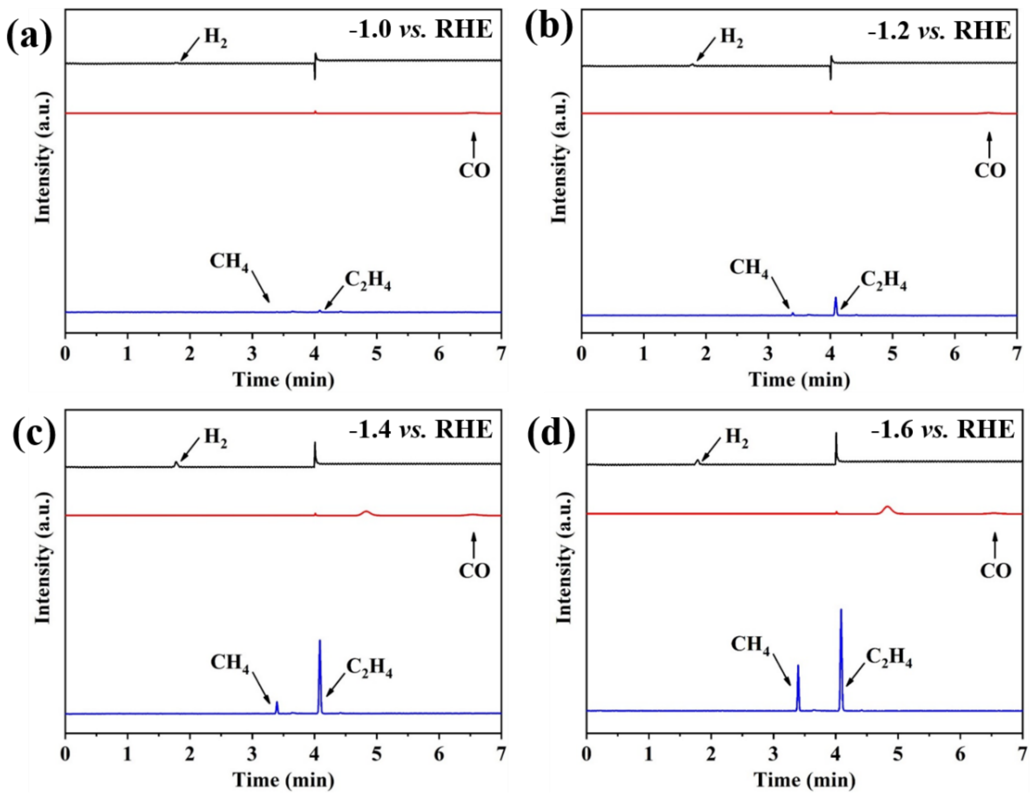

Fig. S18. GC profiles of **Cuophen** after the electrocatalysis at -1.4 V *vs.* RHE for 50 hrs with different potentials: (a) -1.0 *vs.* RHE, (b) -1.2 *vs.* RHE, (c) -1.4 *vs.* RHE and (d) -1.6 *vs.* RHE.

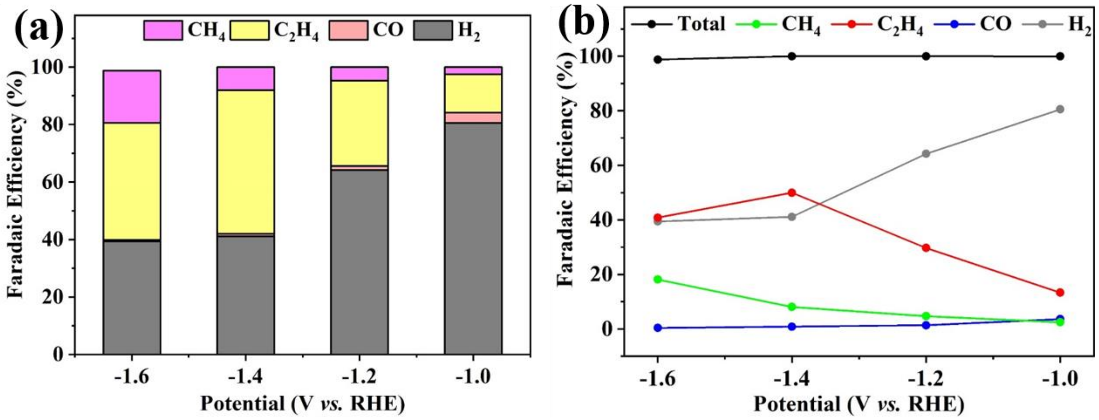

Fig. S19. (a) FEs of CH_4, C_2H_4, CO and H_2 for **Cuophen** after the electrocatalysis. (b) FEs of different reduced products for **Cuophen** after the electrocatalysis at the potentials of -1.0 V to -1.6 V *vs.* RHE.

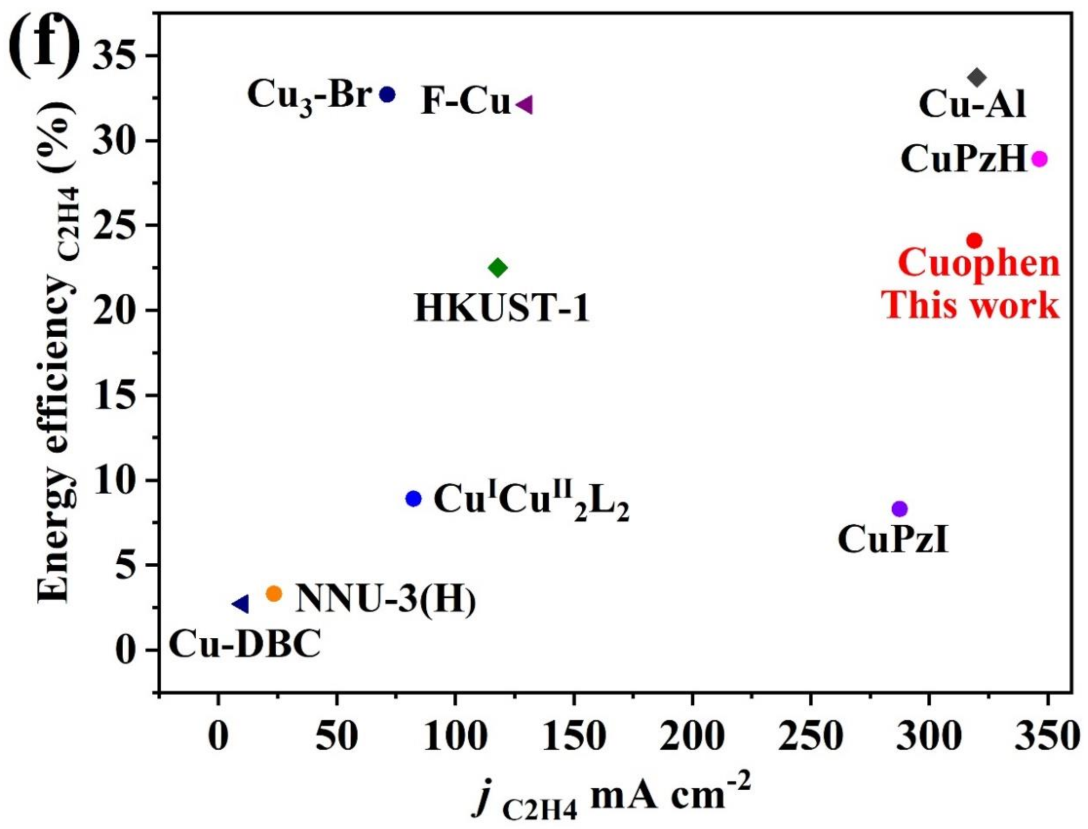

Fig. S20. Energy efficiency as a function of partial current density on **Cuophen** with 1 M KOH in comparison with representative catalysts.

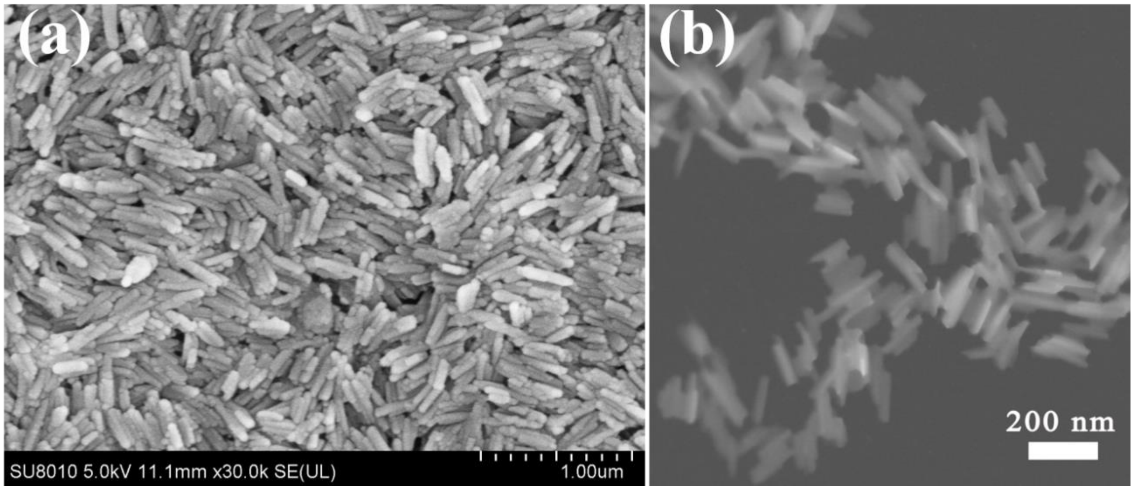

Fig. S21. (a) SEM and (b) TEM images of **Cuophen** after the electrocatalysis.

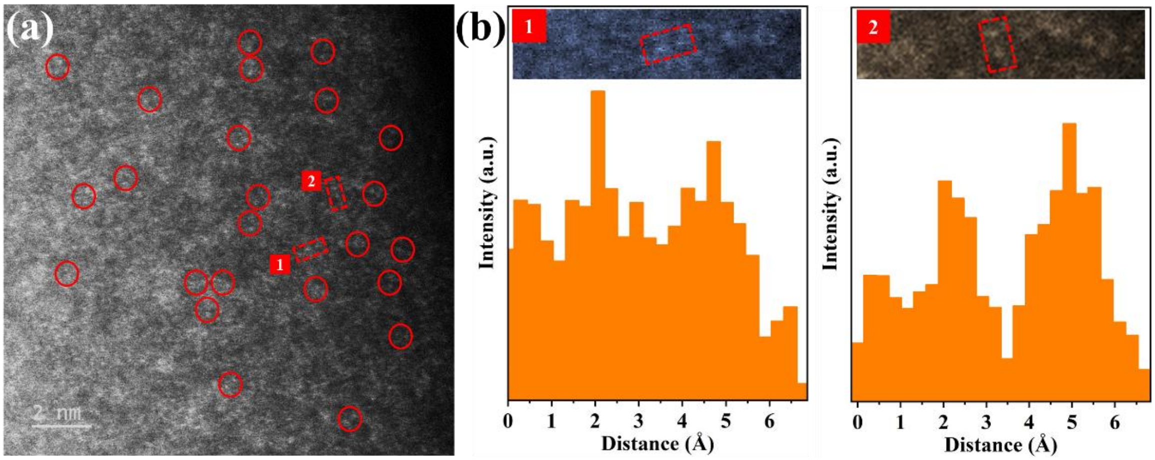

Fig. S22. (a) Aberration-corrected HAADF-STEM image of **Cuophen** after the electrocatalysis. (b) Intensity profiles from the atomic sites 1 and 2 in (a).

Aberration-corrected HAADF-STEM showed that the distance between the two bright spots was not changed (Fig. S9), and no agglomeration was found to be copper clusters, which further indicated that **Cuophen** was stable.

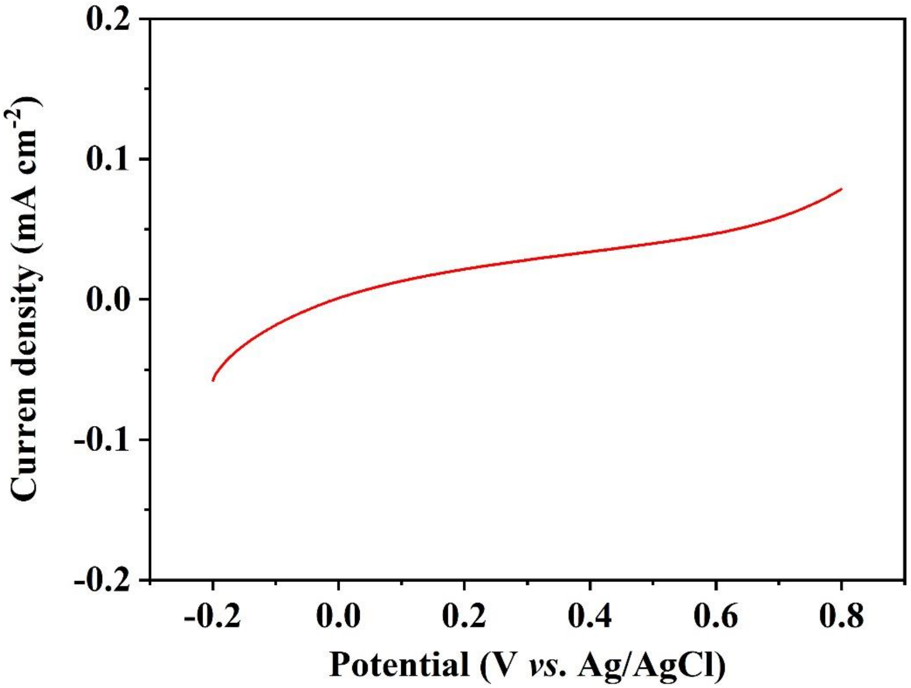

Fig. S23. Anodic stripping voltammograms obtained from **Cuophen**-modfied glassy carbon electrode when the potential was held at -1.4 V *vs.* RHE in a CO_2-saturated 0.1 M $KHCO_3$ aqueous solution (Scan rate: 50 mVs^{-1}).

To rule out the possibility of reduction of the Cu(I) ions into metallic copper and deposition on to the electrode surface, a positive potential was applied on the work electrode after the long-time electrolysis. No redox peak was observed in the LSV curve from -0.2 to 0.8 V *vs*. Ag/AgCl (Figure S23), indicative of the high stability of Cu(I) ions in **Cuophen** during the long-time electrolysis.

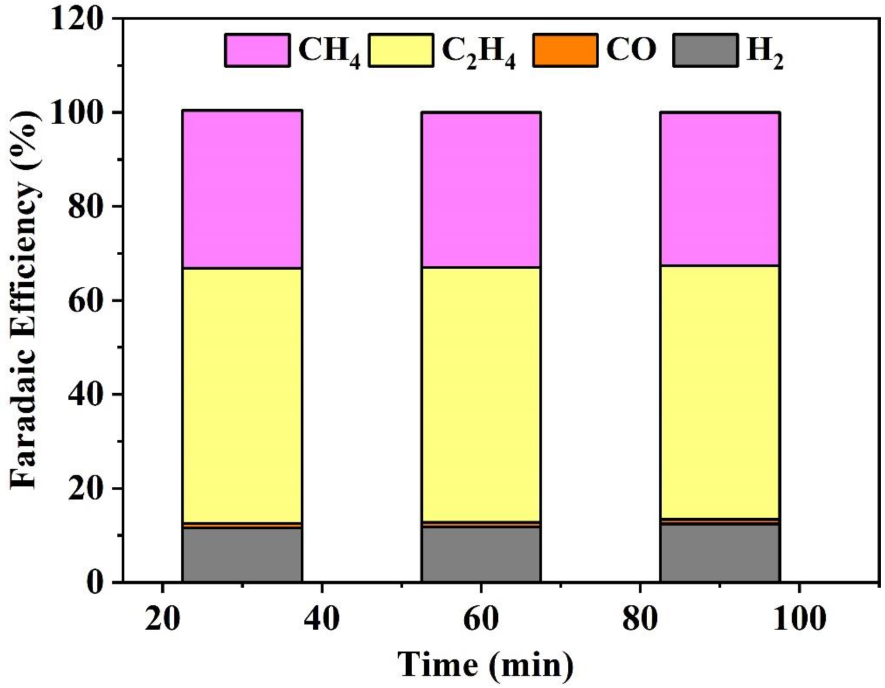

Fig. S24. Faradaic Efficiencies of gaseous products in the first 90 minutes.

We conducted the time-dependent Faradaic Efficiencies of gaseous products and the results (Fig. S24) showed that the selectivities of gas phase products did not change significantly in the first 90 minutes, further demonstrating the stability of **Cuophen** in the eCO_2RR process.

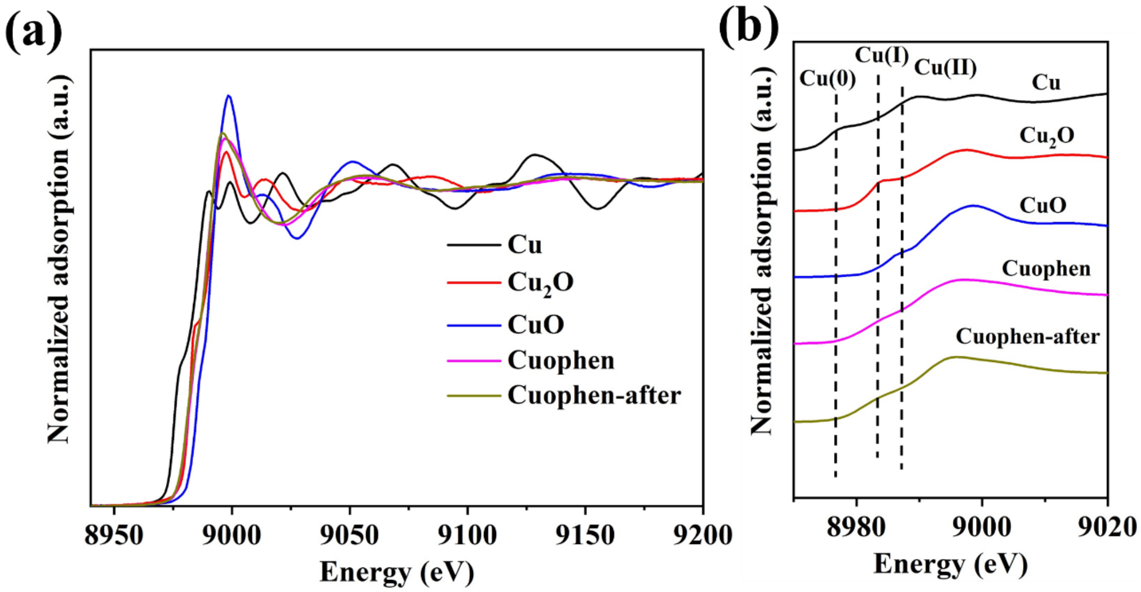

Fig. S25. X-ray absorption spectroscopy characterization of catalyst and references. (a) Cu K-edge *in situ* XANES spectra of the **Cuophen** measured at -1.4 V *vs* RHE in 1 M KOH. (b) Partially magnified *in situ* XANES spectra.

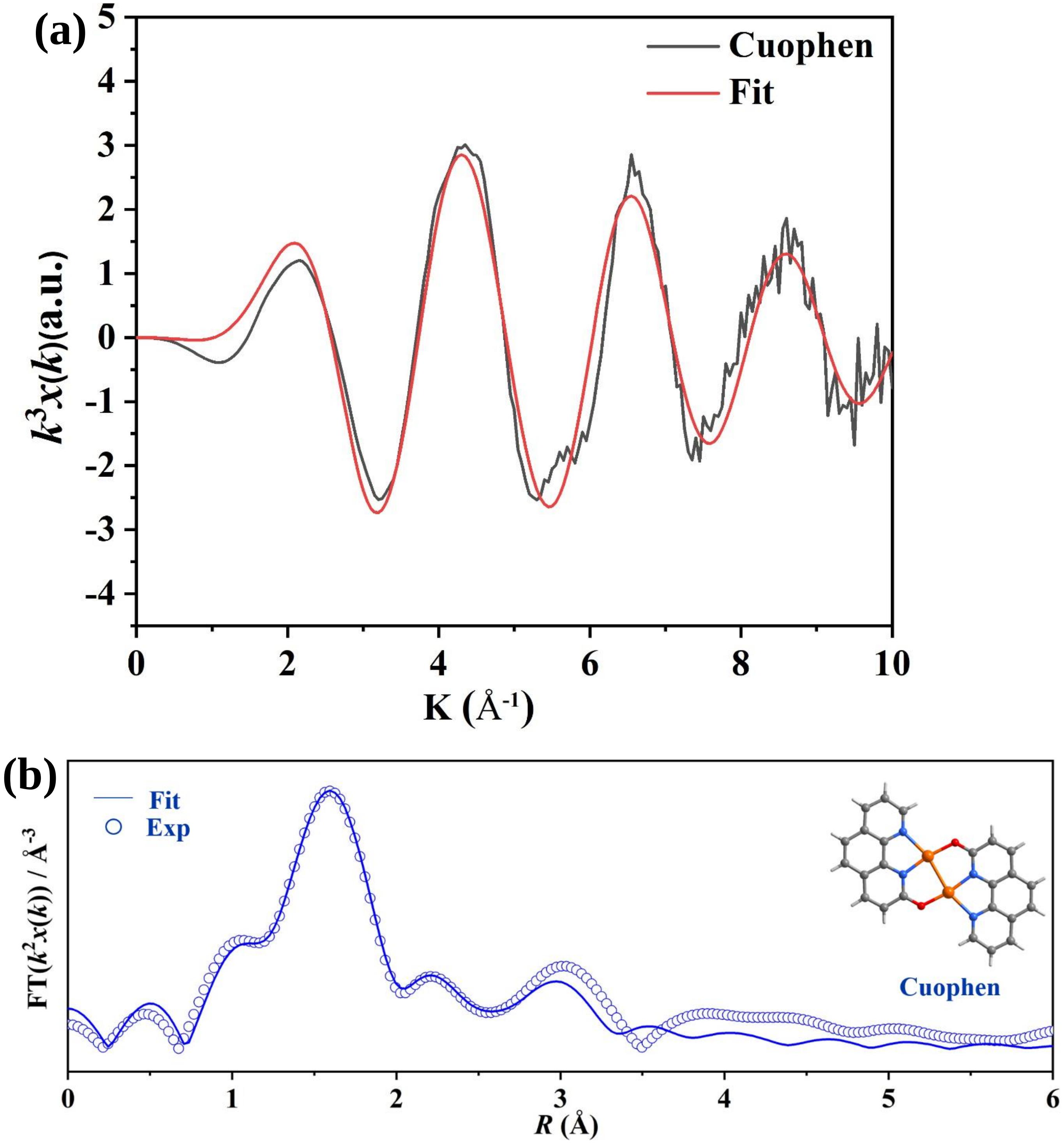
(a)
Cuophen
Fit
$k^3x(k)$(a.u.)
K (Å^{-1})
(b)
Fit
Exp
FT($k^2x(k)$) / Å^{-3}
Cuophen
R (Å)

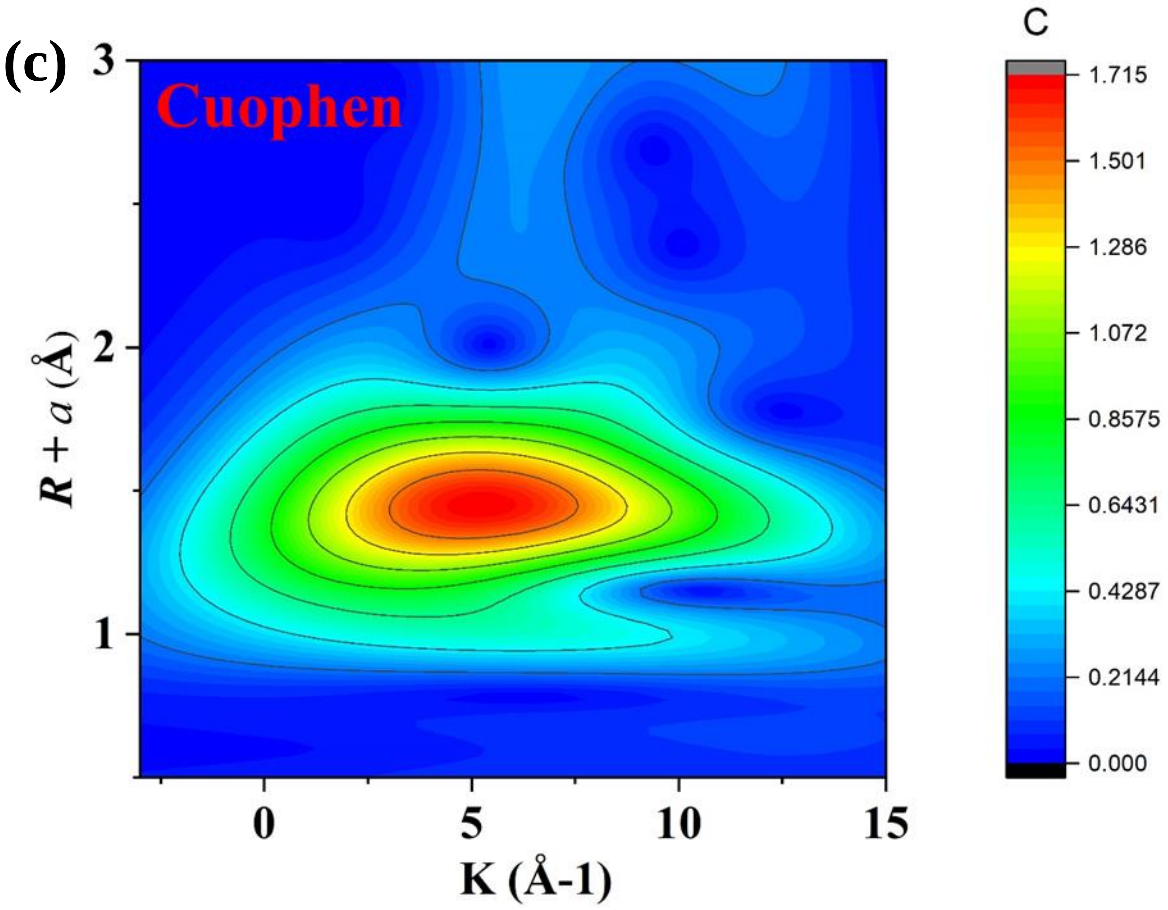

Fig. S26. (a) K' space EXAFS spectrum and fits, (b) R' space EXAFS spectrum and fits and (c) Wavelet transform (WT) contour plots of EXAFS for the **Cuophen** before eCO_2RR.

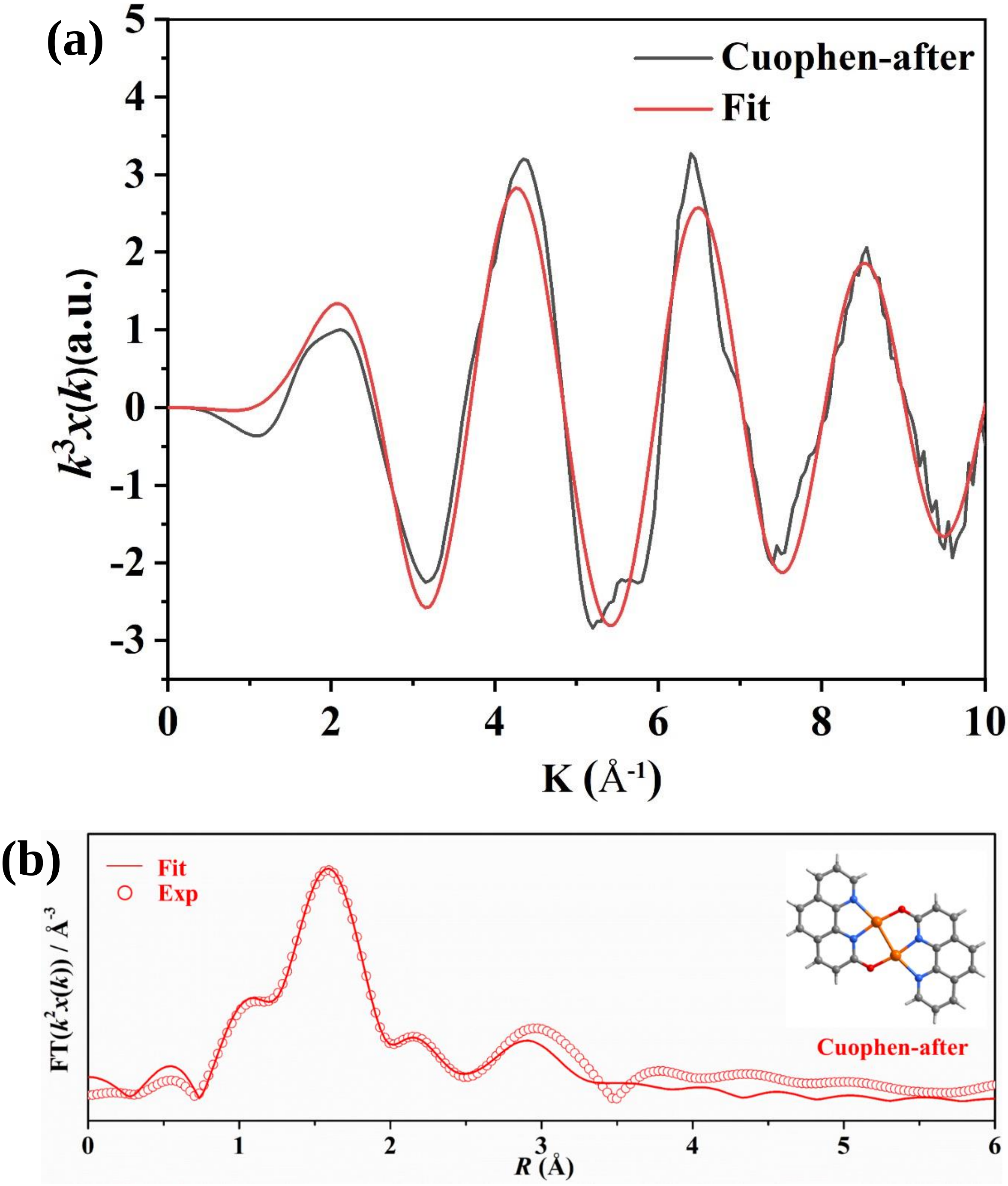
(a)
Cuophen-after
Fit
k3x(k)(a.u.)
K (Å-1)
(b)
Fit
Exp
Cuophen-after
FT(k2x(k)) / Å-3
R (Å)

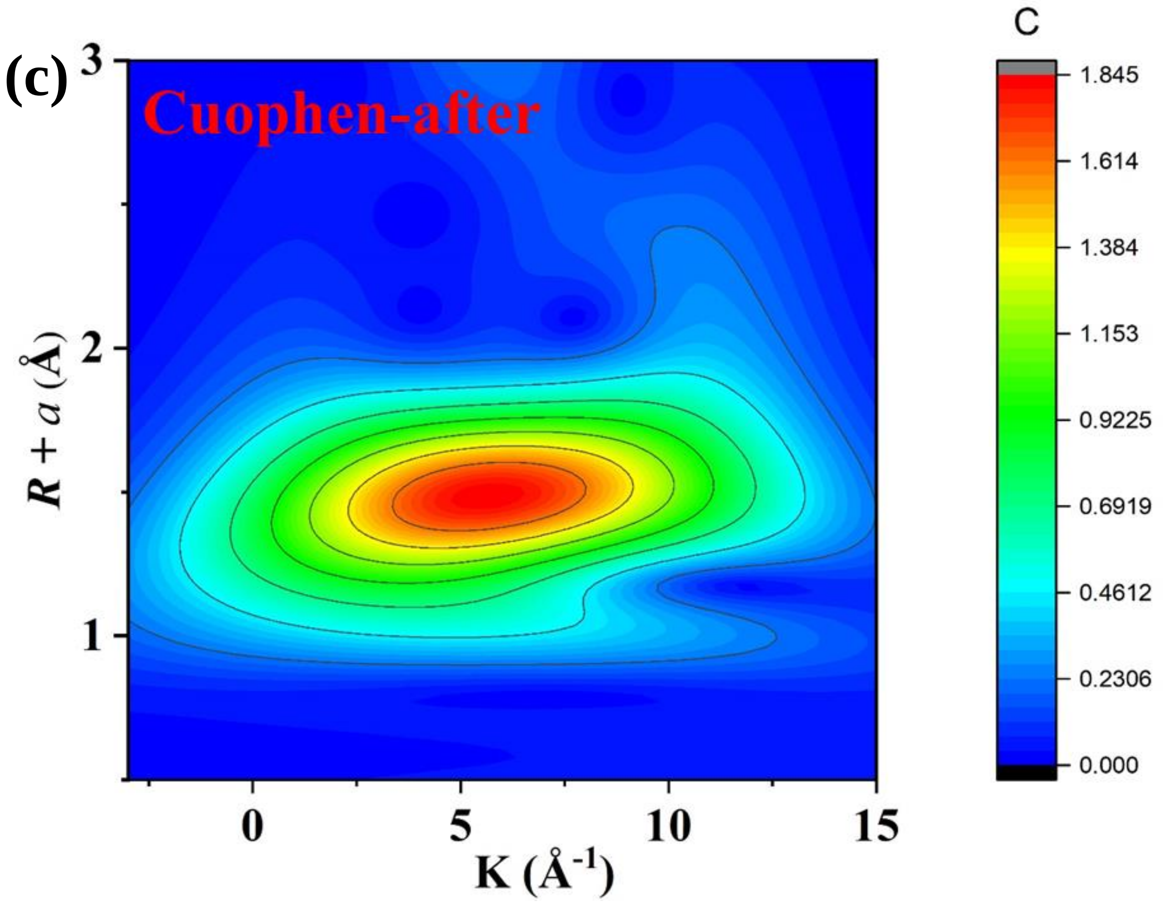

Fig. S27. (a) K' space EXAFS spectrum and fits, (b) R' space EXAFS spectrum and fits and (c) Wavelet transform (WT) contour plots of EXAFS for the **Cuophen** after eCO_2RR.

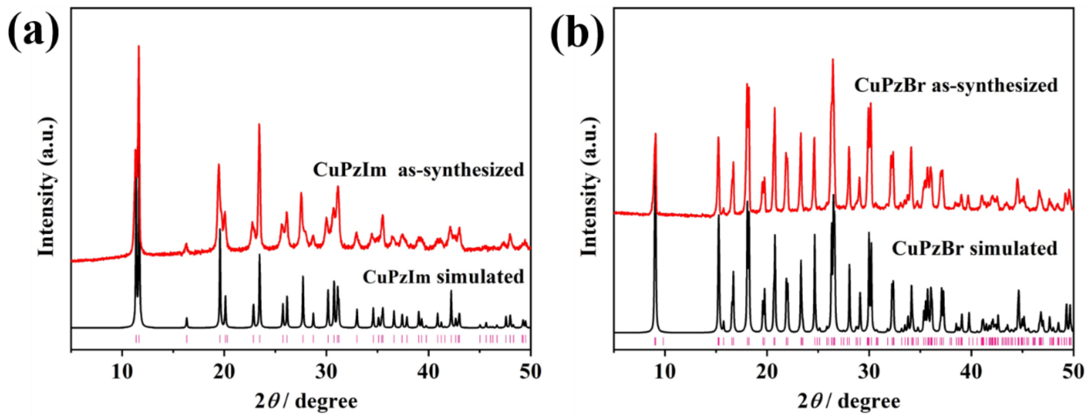

Fig. S28. PXRD patterns of (a) **CuPzIm** and (b) **CuPzBr**.

Table S1. Crystal data and structure refinement for **Cuophen**.

Complex	Cuophen
Formula	$C_{48}H_{28}Cu_4N_8O_4$
Formula weight	1034.96
Temperature (K)	298(2)
Crystal system	monoclinic
Space group	$P2_1/n$
a /Å	10.4377(3)
b /Å	6.0676(2)
c /Å	14.6437(5)
α / °	90
β / °	94.474(3)
γ / °	90
V / Å^3	924.58(6)
Z	4
D_c /g cm^{-3}	1.859
R_{int}	0.0283
R_1 ($I > 2\sigma(I)$)^b	0.0399
wR_2 (all data)^c	0.1147
Completeness / %	99.7
GOF	1.077

^{a} The data are similar yet more precise to those reported by X. M. Zhang, M. L. Tong, M. L. Gong et al., *Chem-Eur. J.*, **2002**, *8*, 3187 – 3194.

^{b} $R_1 = \sum||F_o| - |F_c||/\sum|F_o|$.

^{c} $wR_2 = [\sum w(F_o^2 - F_c^2)^2/\sum w(F_o^2)^2]^{1/2}$.

Table S2. Comparison of the electrocatalytic performances of representative Cu-based catalysts. (Blue: non-network complexes, yellow: inorganic solid, orange: MOFs).

Catalyst	E (V *vs.* RHE)	*J* (mA/cm^2)	C_2H_4(%)	Stability / h	After CO_2RR	Electrolyte	Substrate	Ref.
**Cuophen**	**-1.4**	**52**	**C_2H_4 (55)**	**50**	**Cuophen**	**0.1 M $KHCO_3$**	**Flow cell**	**This work**
		**580**	**CH_4 (33.59)**			**1 M KOH**		
PorCu	-0.98	49	C_2H_4 (17)<br>CH_4 (27)	1	PorCu	0.5 M $KHCO_3$	Flow cell	2
CuPc	-1.06	~19.7	C_2H_4 (4)<br>CH_4 (66)	1	CuPc	0.5 M $KHCO_3$	H-cell	3
{$Cu_3(\mu_3$-OH)(μ-pz)$_3$(Im)$_3$}CBr (**Cu_3-Br**)	-0.7	129.58	C_2H_4 (55.01)	9.5	**Cu_3-Br**	0.5 M KOH	H-cell	4
[Cu(4-PzH)$_2$] (**CuPzH**)	-1.0	577.4	C_2H_4 (60)<br>CH_4 (8.53)	4	**CuPzH**	1 M KOH	Flow cell	5
[Cu(4-PzI)$_2$] (**CuPzI**)	-1.0	552.9	C_2H_4 (16.04)<br>CH_4 (52)	3.5	**CuPzI**	1 M KOH	Flow cell	5
**CuL** (L = 1,1-di-(3-(2-hydroxy-3,5-di-tert-butyl)phenyl)amino)-phenylsulfone)	-1.5	10.3	C_2H_4 (12)<br>CH_4 (70)	6	**CuL**	0.1 M $KHCO_3$	H-cell	6
**$Cu^ICu^{II}_2L_2$** (H_2L = N,N'-(propane-1,3-diyl)bis(1-(1*H*-imidazol-4-yl)methanimine)	-1	477.42	C_2H_4 (17.26)<br>CH_4 (67.76)	1	**$Cu^ICu^{II}_2L_2$**	1 M KOH	Flow cell	7
[Cu(3TPyP)] (**Cu-3TPyP**)	-1	200	CH_4 (62.4)	1	**Cu-3TPyP**	1 M KOH	Flow cell	8
Cu(100)	-1	5	C_2H_4(40.4)<br>CH_4(30.4)	/	Cu(100)	0.1 M $KHCO_3$	Pyrex electrolysis cell	9
**Cu(111)**	-1.15	5	C_2H_4 (8.3)<br>CH_4(46.3)	/	**Cu(111)**	0.1 M $KHCO_3$	Pyrex electrolysis cell	9
Cu	-1.416	225	C_2H_4 (6)<br>CH_4 (48)	20	Cu	1 M $KHCO_3$	Flow cell	10
Cu nanoparticle	-1.1	/	C_2H_4 (36)<br>CH_4(1)	/	Cu	0.1 M $KClO_4$	H-cell	11
polycrystalline Cu	-1.41	5	C_2H_4 (30.1)<br>CH_4 (29.4)	/	Cu	0.1 M $KHCO_3$	H-cell	12
reconstructed Cu	-1.804	18	C_2H_4 (56)<br>CH_4 (5)	/	Cu	0.05 M $KHCO_3$	H-cell	13

Cu NWs	-1.01	17.3	C_2H_4 (77.4)<br>CH_4 (3)	198	Cu NWs	0.1 M $KHCO_3$	H-cell	14
OD-Cu	-0.9	12	C_2H_4 (60)<br>CH_4 (5)	1	Cu-CuO-Cu_2O@Cu	0.1 M $KHCO_3$	H-cell	15
Cu-on-Cu_3N	-1.05	/	C_2H_4 (43)<br>CH_4 (3)	30	Cu	0.1 M $KHCO_3$	H-cell	16
Cu_3N	-1.6	135	C_2H_4 (60)<br>CH_4 (1)	20	Cu_3N	0.1 M $KHCO_3$	Flow cell	17
CuO	-1.95	/	C_2H_4 (~30)<br>CH_4 (~25)	0.5	Cu	0.1 M K_2SO_4	H-cell	18
Cu nanocube-O	-1	~15	C_2H_4 (45)<br>CH_4 (2)	0.25-0.3	Cu	0.1 M $KHCO_3$	H-cell	19
electrodeposited Cu_2O	-0.99	25	C_2H_4 (40.25)<br>CH_4 (2.48)	1.16	Cu	0.1 M $KHCO_3$	H-cell	20
F-Cu	-0.89	1600	C_2H_4 (65)<br>CH_4 (5)	4	Cu(0) / Cu(I)	0.75 M KOH	Flow cell	21
Cu-Al	-1.5	400	C_2H_4 (80)<br>CH_4 (<1)	50	Cu-Al	1 M KOH	Flow cell	22
CuPd nanoalloy	-1.8	/	CH_4 (51)	3	/	0.1 M $KHCO_3$	H-cell	23
Phase-separated CuPd	-0.74	360.5	C_2H_4 (47)	/	/	1 M KOH	Flow cell	40
Cu fiol	-1.0	/	C_2H_4 (22)<br>CH_4 (60)	/	Cu	0.1 M $KHCO_3$+0.3 M KI	H-cell	41
Cu nanowire arrays	-1.1	/	C_2H_4 (17.4)	5	Cu	0.1 M $KClO_4$	H-cell	42
Cu nanocubes	-1.1	5.5	C_2H_4 (41)<br>CH_4 (20.2)	/	Cu	0.1 M $KHCO_3$	H-cell	43
[Cu_2(dptb)]<br>(**NNU-33(H)**)	-0.9	391	C_2H_4 (6.04)<br>CH_4 (82.17)	5	[Cu_2(dptb)]<br>(**NNU-33(H)**)	1 M KOH	Flow cell	24
CuPc-Cu-O	-1.2	7.3	C_2H_4 (50)<br>CH_4 (12)	4	CuPc-Cu-O	0.1 M $KHCO_3$	H-cell	25
$Cu_2[BH(mim)_3]_2Cl_2$<br>(BIF-102)	-1	10	C_2H_4 (11.3)<br>CH_4 (0.54)	5	$Cu_2[BH(mim)_3]_2Cl_2$ (BIF-102)	0.5 M $KHCO_3$	H-cell	26

[Cu_4ZnCl_4(btdd)$_3$] (**Cu_4-MFU-4*l***)	-1.2	9.8	CH_4 (92)	24	[Cu_4ZnCl_4(btdd)$_3$] (**Cu_4-MFU-4*l***)	0.5 M $NaHCO_3$	H-cell	27
[Cu_3(HATNA)$_2$] (**HATNA-Cu**)	-1.5	10.5	C_2H_4 (~3) CH_4 (78)	12	[Cu_3(HATNA)$_2$] (**HATNA-Cu**)	0.1 M $KHCO_3$	H-cell	28
[Cu_2(DBC)] (**Cu-DBC**)	-1.5	203	C_2H_4 (5) CH_4 (~80)	2.5	[Cu_2(DBC)] (**Cu-DBC**)	1 M KOH	Flow cell	29
AuNN@PCN-222(Cu)	-1.2	~7.5	C_2H_4 (~80)	10	AuNN@PCN-222(Cu)	0.1 M $KHCO_3$	H-cell	30
[Cu_3(HITP)$_2$] (**CuHITP**)	-1.56	23.3	C_2H_4 (64)	10	Cu	0.1 M $KHCO_3$	H-cell	31
[Cu_2(BDC)]	-1.3	/	C_2H_4 (~35) CH_4 (0.9)	1	Cu / Cu_2O	0.1 M KI	H-cell	32
[Cu_3(btc)$_2$] (HKUST-1)	-1.07	262	C_2H_4 (45) CH_4 (2)	2.3	Cu clusters	1 M KOH	Flow cell	33
[Cu(ade)(ace)] (**Cu-ade**)	-1.4	8.5	C_2H_4 (45) CH_4 (12)	8	Cu clusters	0.1 M $KHCO_3$	H-cell	34
HKUST-1	-1.16	~16.3	CH_4 (27%)	1	Cu dendritic nanostructures	0.5 M $KHCO_3$	H-cell	3
Cu_2O@HKUST-1	-1.71	~13.3	C_2H_4 (16.2) CH4 (63.2)	1	Cu_2O@HKUST-1	0.1 M $KHCO_3$	H-cell	35
S-HKUST-1	-1.30	20	C_2H_4 (60) CH_4 (5.6)	8	Cu/CuxSy	0.1 M $KHCO_3$	H-cell	36
MAF-2E	-1.30	10.9	C_2H_4 (53.5) CH_4 (21.1)	8	MAF-2E	0.1 M $KHCO_3$	H-cell	37

Table S3. Performance comparison of various catalysts for CO_2 electro-reduction to ethylene in 0.1 M $KHCO_3$.

No.	Catalyst	FE (C_2H_4, %)	*E* (vs. RHE)	*J* (mA/cm^2)	$EE_{half\ cell}$ (C_2H_4)	J_{C2H4} (mA/cm^2)	Ref.
1	**Cuophen**	**55**	**-1.4**	**52**	**24.1**	**28.6**	**This work**
2	PorCu	17	-0.98	49.4	8.8	8.4	2
3	CuPc	4	-1.06	19.7	2.0	13	3
4	Cu(100)	40.4	-1	5	20.8	2.02	9
5	Cu(111)	8.3	-1.15	5	4.0	0.415	9
6	Cu NWs	77.4	-1.01	22.35	39.7	17.3	14
7	CuPc-Cu-O	50	-1.2	7.3	23.7	3.65	25
8	AuNN@PCN-222(Cu)	80	-1.2	7.5	37.9	6	30
9	S-HKUST-1	60	-1.3	20	27.3	12	36
10	MAF-2E	53.3	-1.3	10.9	24.3	5.83	37
11	PTF-(Ni)/Cu	57.3	-1.1	5.5	28.3	3.15	38
12	Re-Cu-I	51	-0.99	22.3	26.4	11 .373	39

Table S4. Performance comparison of various catalysts for CO_2 electro-reduction to ethylene in 1 M KOH. Energy efficiency (EE) is given for the half-cell by assuming no overpotential for the anodic oxygen evolution reaction. Therefore, $EE_{half\ cell}(C_2H_4) = [1.23 + (-E_{C2H4})] * FE_{C2H4} / [1.23 + (-E)]$, where E_{C2H4} is the thermodynamic potential of CO_2RR to ethylene (+0.08 vs. RHE), E is the applied potential versus RHE and FE_{C2H4} denotes as the Faradaic efficiency for ethanol in percentage.

No.	Catalyst	FE (C_2H_4, %)	E (vs. RHE)	J (mA/cm^2)	$EE_{half\ cell}$ (C_2H_4)	J_{C2H4} (mA/cm^2)	Ref.
1	**Cuophen**	**55**	**-1.4**	**580**	**24.1**	**319**	**This work**
2	{$Cu_3(\mu_3$-OH)(μ-pz)$_3$(Im)$_3$}cBr (**Cu_3-Br**)	55	-0.7	129.58	32.7	71.27	4
3	[Cu(4-PzH)$_2$] (**CuPzH**)	60	-1.0	577.4	28.9	346.46	5
4	[Cu(4-PzI)$_2$] (**CuPzI**)	16.04	-1.0	552.9	8.3	287.52	5
5	**Cu'Cu''$_2$L$_2$** (H_2L = N,N -(propane-1,3-diyl)bis(1-(1*H*-imidazol-4-yl)methanimine)	17.26	-1.0	477.42	8.9	82.4	7
6	F-Cu	65	-0.75	200	32.1	130	21
7	Cu-Al	80	-1.5	400	33.7	320	22
8	[Cu$_2$(DBC)] (**Cu-DBC**)	5	-0.9	203	2.7	10.15	32
9	[Cu$_2$(dptb)] (**NNU-33(H)**)	6.04	-0.9	391	3.3	23.6	24
10	[Cu$_3$(btc)$_2$] (**HKUST-1**)	45	-1.07	262	22.5	117.9	3

Table S5. EXAFS fitting parameters of the Cuophen and Cuophen-after sample measured under operando conditions.

Sample	Scattering pair	***R*** **(Å)**	**σ^2 (10^{-3}Å^2)**	**ΔE_0 (eV)**	***R*** **factor**
**Cuophen**	Cu-O	1.98±0.04	4.6	4.17	0.0019
	Cu-N_1	2.01±0.05	4.6	4.17	0.0019
	Cu-N_2	2.29±0.01	4.6	4.17	0.0019
	Cu-Cu	2.68±0.06	4.6	4.17	0.0019

Sample	Scattering pair	***R*** **(Å)**	**σ^2 (10^{-3}Å^2)**	**ΔE_0 (eV)**	***R*** **factor**
**Cuophen-after**	Cu-O	1.99±0.07	5.9	-0.32	0.0018
	Cu-N_1	2.01±0.05	5.9	-0.32	0.0018
	Cu-N_2	1.96±0.13	5.9	-0.32	0.0018
	Cu-Cu	2.73±0.11	5.9	-0.32	0.0018

Note: S_0^2 is the amplitude reduction factor; CN is the coordination number = 1.0; R is interatomic distance (the bond length between the central atoms and surrounding coordination atoms); σ^2 is Debye-Waller factor (a measure of thermal and static disorder in absorber-scattered distances); ΔE_0 is edge-energy shift (the difference between the zero kinetic energy value of the sample and that of the theoretical model). *R* factor is used to value the goodness of the fitting.

SI References

[1] J. P. Zhang, Y. B. Wang, X. C. Huang et al., “Metallophilicity Versus π - π Interactions: Ligand-Unsupported Argentophilicity/Cuprophilicity in Oligomers-of-Dimers $[M_2L_2]_n$ (M=Cu^I or Ag^I, L=tridentate ligand),” *Chemistry - A European Journal,* vol. 11, no. 2, pp. 552-561, 2005.

[2] Z. Weng, J. Jiang, Y. Wu, Z. Wu et al., “Electrochemical CO_2 Reduction to Hydrocarbons on a Heterogeneous Molecular Cu Catalyst in Aqueous Solution,” *Journal of the American Chemical Society*, vol. 138, no. 26, pp. 8076-8079, 2016.

[3] Z. Weng, Y. Wu, M. Wang et al., “Active Sites of Copper-Complex Catalytic Materials for Electrochemical Carbon Dioxide Reduction,” *Nature Communications,* vol. 9, no. 1, pp. 415-424, 2018.

[4] Y. F. Lu, L. Z. Dong, J. Liu et al., “Predesign of Catalytically Active Sites via Stable Coordination Cluster Model System for Electroreduction of CO_2 to Ethylene,” *Angewandte Chemie International Edition,* vol. 60, no. 50, pp. 26210-26217, 2021.

[5] R. Wang, J. Liu, Q. Huang et al., “Partial Coordination-Perturbed Bi-Copper Sites for Selective Electroreduction of CO_2 to Hydrocarbons,” A*ngewandte Chemie International Edition*, vol. 60, no. 36, pp. 19829-19835, 2021.

[6] X. Wang, Z. W. Li, Z. J. Huang et al., “Boosting CH_4 Selectivity in CO_2 Electroreduction Using a Metallacycle-Based Porous Crystal with Biomimetic Adaptive Cavities,” *Journal of Materials Chemistry A,* vol. 10, no. 22, pp. 1948-11954, 2022.

[7] J. N. Lu, J. Liu, L. Zhang et al., “Crystalline Mixed-Valence Copper Supramolecular Isomers for Electroreduction of CO_2 to Hydrocarbons,” *Journal of Materials Chemistry A*, vol. 9, no. 41, pp. 23477-23484, 2021.

[8] S. N. Sun, J. N. Lu, Q. Li et al., “Establishing Spatially Elastic Hydrogen-Bonding Interaction in Electrochemical Process for Selective CO_2-to-CH_4 Conversion,” *Chem Catalysis*, vol. 1, no. 5, pp. 1133-1144, 2021.

[9] Y. Hori, I. Takahashi, O. Koga, and N. Hoshi, “Electrochemical Reduction of Carbon Dioxide at Various Series of Copper Single Crystal Electrodes,” *Journal of Molecular Catalysis A: Chemical*, vol. 199, no. 1, pp. 39-47, 2003.

[10] X. Wang, A. Xu, F. Li et al., “Efficient Methane Electrosynthesis Enabled by Tuning Local CO_2 Availability,” *Journal of the American Chemical Society*, vol. 142, no. 7, pp. 3525-3531, 2020.

[11] W. Tang, A. Peterson, A. Varela et al., “The Importance of Surface Morphology in Controlling the Selectivity of Polycrystalline Copper for CO_2 Electroreduction,” *Physical Chemistry Chemical Physics*, vol. 14, no. 1, pp. 76-81, 2012.

[12] H. Yoshio, M. Akira, and T. Ryutaro, “Formation of Hydrocarbons in the Electrochemical Reduction of Carbon Dioxide at a Copper Electrode in Aqueous Solution,” *Journal of the Chemical Society, Faraday Transactions*, vol. 85, no. 8, pp. 2309-2326, 1989.

[13] M. Kibria, C. T. Dinh, A. Seifitokaldani et al., “A Surface Reconstruction Route to High Productivity and Selectivity in CO_2 Electroreduction toward C_{2+} Hydrocarbons,” *Advanced Materials*, vol. 30, no. 49, 1804867, 2018.

[14] C. Choi, S. Kwon, T. Cheng et al., "Highly Active and Stable Stepped Cu Surface for Enhanced Electrochemical CO_2 Reduction to C_2H_4," *Nature Catalysis*, vol. 3, no. 10, pp. 804-812, 2020.

[15] H. Mistry, A. Varela, C. Bonifacio et al., "Highly Selective Plasma-Activated Copper Catalysts for Carbon Dioxide Reduction to Ethylene," *Nature Communications*, vol. 7, no. 1, pp. 1-9, 2016.

[16] Z. Q. Liang, T. T. Zhuang, A. Seifitokaldani et al., "Copper-on-nitride Enhances the Stable Electrosynthesis of Multi-Carbon Products from CO_2," *Nature Communications*, vol. 9, no. 1, pp. 1-8, 2018.

[17] Z. Yin, C. Yu, Z. Zhao et al., "Cu_3N Nanocubes for Selective Electrochemical Reduction of CO_2 to Ethylene," *Nano Letters*, vol. 19, no. 12, pp. 8658, 2019.

[18] J. Yano, S. Yamasaki, "Pulse-Mode Electrochemical Reduction of Carbon Dioxide Using Copper and Copper Oxide Electrodes for Selective Ethylene Formation," *Journal of Applied Electrochemistry*, vol. 38, no. 12, pp. 1721-1726, 2008.

[19] D. Gao, I. Zegkinoglou, N. Divins et al., "Plasma-Activated Copper Nanocube Catalysts for Efficient Carbon Dioxide Electroreduction to Hydrocarbons and Alcohols," *ACS Nano*, vol. 11, no. 5, pp. 4825-4831, 2017.

[20] D. Ren, Y. Deng, A. Handoko et al., "Selective Electrochemical Reduction of Carbon Dioxide to Ethylene and Ethanol on Copper(I) Oxide Catalysts," *ACS Catalysis*, vol. 5, no. 5, pp. 2814-2821, 2015.

[21] W. Ma, S. Xie, T. Liu et al., "Electrocatalytic Reduction of CO_2 to Ethylene and Ethanol through Hydrogen-Assisted C-C Coupling over Fluorine-Modified Copper," *Nature Catalysis*, vol. 3, no. 6, pp. 478-487, 2020.

[22] M. Zhong, K. Tran, Y. Min et al., "Accelerated Discovery of CO_2 Electrocatalysts Using Active Machine Learning," *Nature*, vol. 581, pp. 178-183, 2020.

[23] S. Zhang, P. Kang, M. Bakir et al., "Polymer-Supported CuPd Nanoalloy as a Synergistic Catalyst for Electrocatalytic Reduction of Carbon Dioxide to Methane," *Proceedings of the National Academy of Sciences of the United States of America*, vol. 112, no. 52, pp. 15809-15814, 2015.

[24] L. Zhang, X. X. Li, Z. L. Lang et al., "Enhanced Cuprophilic Interactions in Crystalline Catalysts Facilitate the Highly Selective Electroreduction of CO_2 to CH_4," *Journal of the American Chemical Society*, vol.143, no. 10, pp. 3808-3816, 2021.

[25] X. F. Qiu, H. L. Zhu, J. R. Huang et al., "Highly Selective CO_2 Electroreduction to C_2H_4 Using a Metal-Organic Framework with Dual Active Sites," *Journal of the American Chemical Society*, vol. 143, no. 19, pp. 7242-7246, 2021.

[26] P. Shao, W. Zhou, Q. L. Hong et al., "Synthesis of a Boron-Imidazolate Framework Nanosheet with Dimer Copper Units for CO_2 Electroreduction to Ethylene," *Angewandte Chemie International Edition*, vol. 60, no. 30, pp. 16687-16692, 2021.

[27] H. L. Zhu, J. R. Huang, X. W. Zhang et al., "Highly Efficient Electroconversion of CO_2 into CH_4 by a Metal-Organic Framework with Trigonal Pyramidal $Cu(I)N_3$ Active Sites," *ACS Catalysis*, vol. 11, no. 18, pp. 11786-11792, 2021.

[28] Y. Liu, S. Li, L. Dai et al., "The Synthesis of Hexaazatrinaphthylene-Based 2D Conjugated Copper Metal-Organic Framework for Highly Selective and Stable

Electroreduction of CO_2 to Methane," *Angewandte Chemie International Edition*, vol. 60, no. 30, pp. 16409-16415, 2021.

[29] Y. Zhang, L. Z. Dong, S. Li et al., "Coordination Environment Dependent Selectivity of Single-Site-Cu Enriched Crystalline Porous Catalysts in CO_2 Reduction to CH_4," *Nature Communications*, vol. 12, no. 6390, pp. 1-9, 2021.

[30] X. Xie, X. Zhang, M. Xie et al., "Au-Activated N Motifs in Non-Coherent Cupric Porphyrin Metal Organic Frameworks for Promoting and Stabilizing Ethylene Production," *Nature Communications*, vol.13, no. 63, pp. 1-11, 2022.

[31] H. Sun, L. Chen, L. Xiong et al., "Promoting Ethylene Production over a Wide Potential Window on Cu Crystallites Induced and Stabilized Via Current Shock and Charge Delocalization," *Nature Communications*, vol. 12, no. 6823, pp. 1-11, 2021.

[32] X. Zhou, J. Dong, Y. Zhu et al., "Molecular Scalpel to Chemically Cleave Metal-Organic Frameworks for Induced Phase Transition," *Journal of the American Chemical Society*, vol. 143, no. 17, pp. 6681-6690, 2021.

[33] D. H. Nam, O. Bushuyev, J. Li et al., "Metal-Organic Frameworks Mediate Cu Coordination for Selective CO2 Electroreduction," *Journal of the American Chemical Society*, vol. 140, no. 36, pp. 11378-11386, 2018.

[34] F. Yang, A. Chen, P. Deng et al., "Highly Efficient Electroconversion of Carbon Dioxide into Hydrocarbons by Cathodized Copper-Organic Frameworks," *Chemical Science*, vol. 10, no. 34, pp. 7975-7981, 2019.

[35] X. Tan, C. Yu, C. Zhao et al., "Restructuring of Cu_2O to Cu_2O@Cu-Metal-Organic Frameworks for Selective Electrochemical Reduction of CO_2," *ACS Applied Materials & Interfaces*, vol. 11, no. 10, pp. 9904-9910, 2019.

[36] C. Wen, M. Zhou, P. Liu et al., "Highly Ethylene-Selective Electrocatalytic CO_2 Reduction Enabled by Isolated Cu-S Motifs in Metal-Organic Framework Based Precatalysts," *Angewandte Chemie International Edition*, vol. 61, no. 2, pp. 1-8, 2022.

[37] L. L. Zhuo, P. Chen, K. Zheng et al., "Flexible Cuprous Triazolate Frameworks as Highly Stable and Efficient Electrocatalysts for CO_2 Reduction with Tunable C_2H_4/CH_4 Selectivity," *Angewandte Chemie International Edition,* Article ID: 10.1002/anie.202204967, 2022.

[38] D. L. Meng, M. D. Zhang, D. H. Si et al., "Highly Selective Tandem Electroreduction of CO_2 to Ethylene over Atomically Isolated Nickel-Nitrogen Site/Copper Nanoparticle Catalysts," *Angewandte Chemie International Edition*, vol. 133, no. 48, pp. 25485-25492, 2021.

[39] J. Han, C. Long, J. Zhang et al., "A Reconstructed Porous Copper Surface Promotes Selectivity and Efficiency toward C_2 Products by Electrocatalytic CO_2 Reduction," *Chemical Science*, vol. 11, no. 39, pp. 10698-10704, 2020.

[40] A. S. Varela, W. Ju, T. Reier, and P. Strasser, "Tuning the Catalytic Activity and Selectivity of Cu for CO_2 Electroreduction in the Presence of Halides," *ACS Catalysis*, vol. 6, no. 4, pp. 2136-2144, 2016.

[41] S. Ma, M. Sadakiyo, M. Heima et al., "Electroreduction of Carbon Dioxide to Hydrocarbons Using Bimetallic Cu-Pd Catalysts with Different Mixing Patterns," *Journal of the American Chemical Society*, vol. 139, no. 1, pp. 47-50, 2017.

[42] M. Ma, K. Djanashvili, and W. A. Smith., "Controllable Hydrocarbon Formation from the Electrochemical Reduction of CO_2 over Cu Nanowire Arrays," *Angewandte Chemie International Edition*, vol. 55, no. 23, pp. 6680-6684, 2016.

[43] A. Loiudice, P. Lobaccaro, E. A. Kamali et al., "Tailoring Copper Nanocrystals towards C_2 Products in Electrochemical CO_2 Reduction," *Angewandte Chemie International Edition*, vol. 55, no. 19, pp. 5789-5792, 2016.
